# Evidence of diverse animal exploitation during the Middle Paleolithic at Ghar-e Boof (southern Zagros)

**DOI:** 10.1038/s41598-023-45974-8

**Published:** 2023-11-03

**Authors:** Mario Mata-González, Britt M. Starkovich, Mohsen Zeidi, Nicholas J. Conard

**Affiliations:** 1 Institute for Archaeological Sciences, University of Tübingen, Hölderlinstr. 12, 72074 Tübingen, Germany; 2 Department of Anthropology, University of Connecticut, Unit 1176, 354 Mansfield Road, Storrs, CT 06269 USA; 3 Senckenberg Centre for Human Evolution and Paleoenvironment (SHEP), Hölderlinstr. 12, 72074 Tübingen, Germany; 4 Department of Early Prehistory and Quaternary Ecology, University of Tübingen, Schloss Hohentübingen, 72070 Tübingen, Germany

**Keywords:** Ecology, Evolution

## Abstract

Although Middle Paleolithic (MP) hominin diets consisted mainly of ungulates, increasing evidence demonstrates that hominins at least occasionally consumed tortoises, birds, leporids, fish, and carnivores. Until now, the MP zooarchaeological record in the Zagros Mountains has been almost exclusively restricted to ungulates. The narrow range of hominin prey may reflect socioeconomic decisions and/or environmental constraints, but could also result from a research bias favoring the study of large prey, since archaeologists have undertaken no systematic taphonomic analyses of small game or carnivores in the region. Here, we report on the first comprehensive taphonomic analysis of an MP faunal assemblage from Ghar-e Boof (∼ 81–45 kyr), a Late Pleistocene site in the southern Zagros of Iran. Anthropogenic bone surface modifications point to hominins as the main agent of accumulation. Hominins preyed primarily on ungulates, particularly wild goat. However, we also found evidence for MP hominin exploitation of carnivores and tortoises at the site. Although small game represents only a minor portion of the diet, our results suggest that the hunting behavior of MP hominins in the Zagros was more diverse than previously thought, similar to what we find elsewhere in Eurasia.

## Introduction

The reconstruction of past hominin diets and subsistence strategies constitutes one of the primary goals of zooarchaeological studies since it informs us about how hominins adapted to and interacted with different environments. There is a general consensus among archaeologists that the animal component of the diet and foraging spectrum of Neanderthals and other Middle Paleolithic (MP) hominins was mainly dominated by ungulates or large game species across most of Eurasia^1–10^. Nevertheless, there has been an increasing body of evidence that demonstrates the hominin exploitation of tortoises^10–15^, birds^16–21^, leporids^22–25^, fish^26,27^, and small and large carnivores^21,28,29^ during the MP (for a more detailed synthesis of the available evidence and latest updates, see^30^ and references therein). Independently if small game and carnivore taxa were systematically^18–20,22,24^ or sporadically^15,21,23,28^ collected or exploited when MP hominins encountered them while foraging, their presence in the zooarchaeological record allows us to better understand and assess crucial aspects of hominin socioeconomics, behavioral variability, and hunting capabilities.

Within the prey choice model of optimal foraging theory, small game is normally considered low-ranked prey in comparison to high-ranked, large game^5,11,12^ (but see^31^). However, small animals with low capture or handling costs, such as slow-moving tortoises or sessile shellfish, have higher net yields relative to small, fast-moving game^5,11,12^. As a result, tortoises might have represented higher-ranked and easy-to-catch resources collected by foragers of different ages and sexes^32,33^. On the other hand, leporids, birds, and fish are quick, and more difficult-to-catch animals, that generally have lower caloric yields^5,11,12^. Moreover, economic decisions to include small game in the diet can be related to environmental constraints and prey availability^22,25,30^, which directly affects the encounter rate^34^. From a technological standpoint, hunting small, fast-moving game may require the use of trapping techniques, such as nets and snares^19,25^, or more efficient procurement methods (e.g., mass collecting^31,35^), which, in turn, can reduce capture costs and increase overall return rates^11,12,31,35^.

Besides the dietary use of small game^19,20,22,23^, archaeologists have suggested that MP hominins might have also exploited rabbits for pelts^25^ and birds for feathers^16–18,20^, the latter of which has been interpreted as an indicator of symbolic behavior. Since both large carnivores and MP hominins were top predators within the ecosystems that they lived, the documentation of carnivore remains in the zooarchaeological record can shed light on the interspecific competition for food resources, landscape and space use, and predatory-prey relationships^36,37^. Cut-marked carnivore bones associated with defleshing and skinning activities indicate the active exploitation of carnivores by MP hominins, not only for the acquisition of food but also fur^21,28,29^.

**Figure 1:**
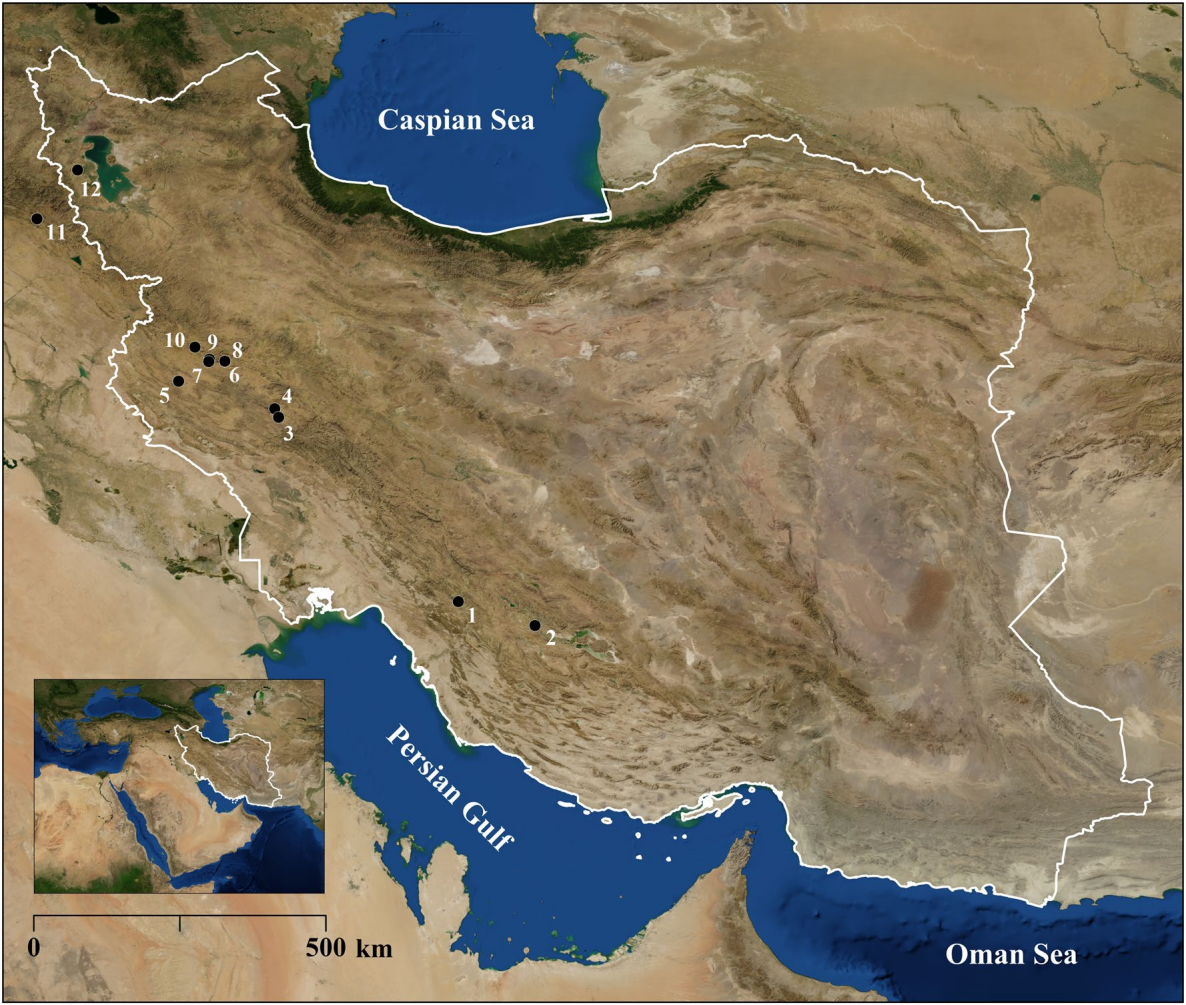
Location of Ghar-e Boof (1) in Southwest Asia and in the Zagros region, along with other Late Pleistocene sites with MP sequences mentioned in the text: Eshkaft-e Gavi (2), Kunji Cave (3), Kaldar Cave (4), Wezmeh Cave (5), Bisotun Cave (6), Warwasi Rockshelter (7), Ghar-e Khar (8), Kobeh Cave (9), Bawa Yawan Rockshelter (10), Shanidar Cave (11), Tamtama Cave (12). Map created by QGIS 3.10.12 (https://www.qgis.org/).

The Zagros Mountains represent a key geographic region in southwestern Asia for the study of human evolution and cultural and behavioral adaptations during the MP, especially because of their heterogeneous topography and high environmental diversity^38^. However, although important archaeological sites in the Zagros have yielded animal bones in direct association with lithic artifacts or even hominin remains (Fig. 1), up until now the MP zooarchaeological record in the region is almost exclusively restricted to ungulate species^4,39–49^. The only exception is Shanidar Cave, where Evins^42^ proposed that land tortoise might have had an important supplementary economic value for hominins, based on the continuous occurrence of this taxon throughout the MP sequence, its higher frequency in relation to other species, and the presence of burned shell fragments. Overall, the presumably narrow range of hominin prey deduced from previous studies^42,43,47,48^ may reflect socioeconomic decisions (e.g., a focus on high-ranked, large game to maximize energetic returns^5,11,12^). Alternatively, some scholars suggest that in the Zagros Mountains the narrow exploitation of ungulate taxa, mostly caprines, was due to environmental constraints^48^. Nevertheless, the predominance of ungulates in MP zooarchaeological assemblages could also be the result of research bias, caused by a disproportionate study of large prey by zooarchaeologists^4,43–46,48,49^, especially since zooarchaeologists have undertaken no systematic taphonomic analyses of small game nor carnivores in the Zagros region. Therefore, further investigations are still required.

Here, we present the first results of a comprehensive taphonomic analysis of the MP faunal assemblage from Ghar-e Boof, dated between ca. 81–45 kyr (OSL dates^50^). The main goals of our study are: (1) to determine whether hominins were the primary agents of bone accumulation or modification at the site, and identify other post-depositional processes that might have affected and altered the preservation of the zooarchaeological remains; and (2) to reconstruct and evaluate hominin prey choice and subsistence strategies during the MP. In this paper, we not only confirm that hominins were the main accumulation agent, but we also report direct evidence for MP hominin exploitation of carnivores and tortoises in the region. Although caprines represented the main prey and food resources exploited at Ghar-e Boof, we demonstrate that the hunting behavior of MP hominins in the Zagros was more diverse than previously thought.

## Geographical, archaeological, and chronological setting

**Figure 2:**
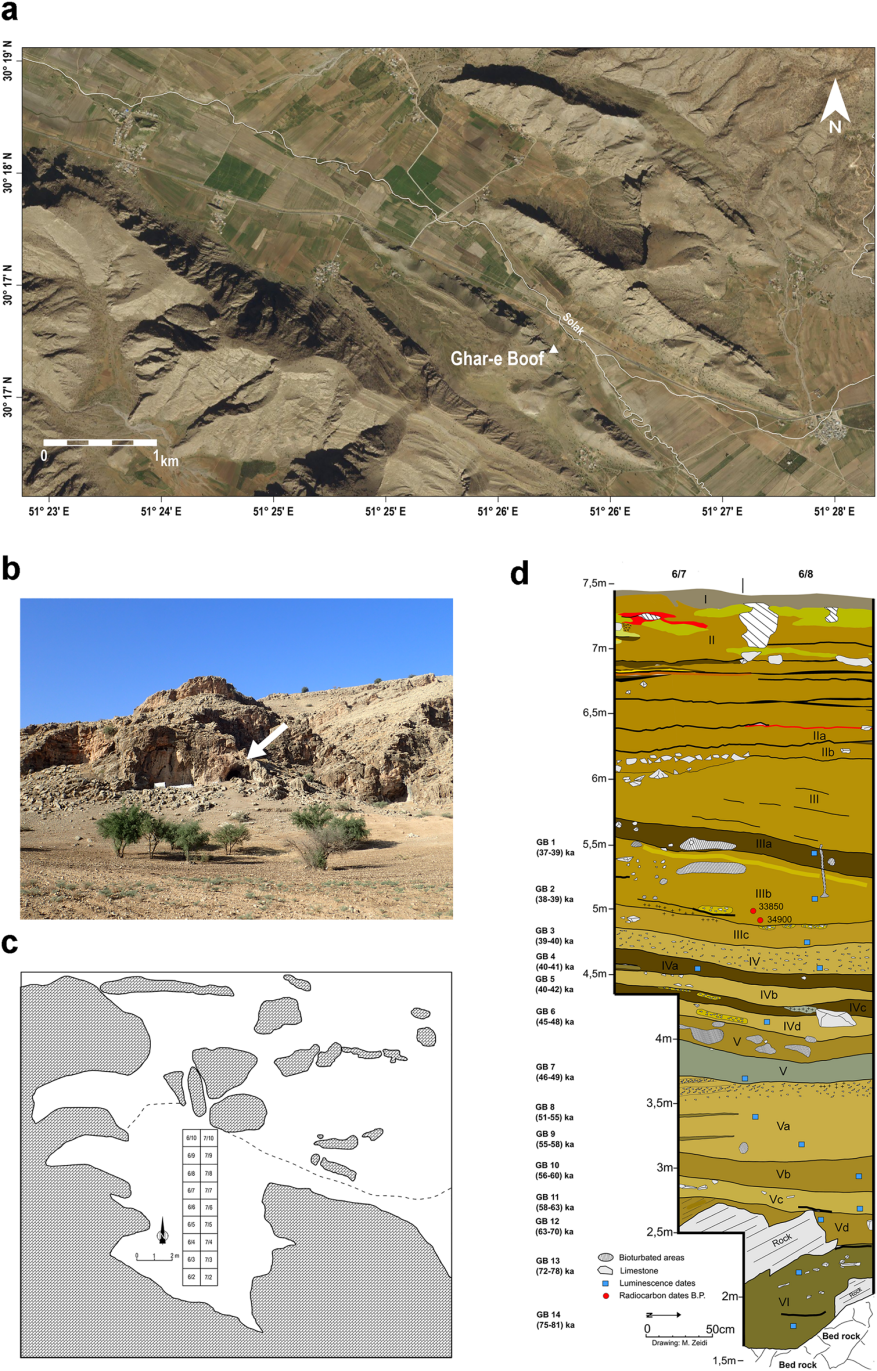
Ghar-e Boof: (**a**) location of the cave in the Dasht-e Rostam region, Iran (satellite view; map created by QGIS 3.10.12, https://www.qgis.org/); (**b**) general view of the entrance of the site (white arrow); (**c**) Schematic representation of the interior of the cave and location of the excavation area divided by quadrants (created by Inkscape 0.92.4, https://inkscape.org; and modified from^53^; dotted line indicates the dripline); (**d**) chrono-stratigraphic sequence (west profile; some of the OSL dates were obtained for the north profile, see^50^, but we included them here at approximate locations within the west profile in order to offer a comprehensive view of the chronology).

Ghar-e Boof (N 30.2839°, E 51.4352°) is located in the Dasht-e Rostam region, in the northwest of Fars Province (southern Zagros Mountains, Iran, Fig. 2a). The topography of the region is heterogenous, with mountains ranging between 700 to 2500 m.a.s.l., and numerous plains and river valleys, which represent natural east–west and north–south corridors^51,52^. Formed in limestone and with an area of about 60 m^2^, the cave lies at an altitude of 905 m.a.s.l., and its entrance faces north^53^ (Fig. 2b). The valley bottom is currently situated approximately 190 m directly downslope from Ghar-e Boof, and a seasonal stream, the Solak River, runs ~ 200 m away towards the northeast. Ghar-e Boof was discovered in 1997 by R. Nowroozi, a member of the Fars cultural heritage office^54^, though the site was originally named Eshkaft-e Yagheh Sangar. The Tübingen-Iranian Stone Age Research Project Team visited Ghar-e Boof in 2005 for the first time, documenting and collecting numerous lithic artifacts on the surface of the site, and assessing its archaeological potential^55,56^. The first excavations were carried out in 2006 and 2007, co-directed by N. J. Conard and M. Zeidi^57^, while another two seasons of excavation took place in 2015 and 2017^58,59^.

The excavation at Ghar-e Boof has an area of 18 m^2^ (2 by 9 m), extending from near the entrance of the site to the back wall, across its north–south axis^53^ (Fig. 2c). The overall stratigraphic sequence consists of ca. 6 m of well-stratified deposits^50^, characterized mostly by ashy silts and silty sediments with limestone clasts of different sizes. These sediments derive primarily from aeolian processes and the effects of gravity, which includes rocks and silts detached from the cave walls and roof^60^. The hominin occupation of the site spans from the MP or MIS5a to the historical period (Table S1^61^, and references therein).

Archaeologists identified six main geological and archaeological horizons (AH)s, and 13 sub-horizons (Fig. 2d). Moving from the top downward, the stratigraphic sequence begins with Holocene deposits (AHs I and II). In these layers, pottery sherds, metal, and glass artifacts from historical periods were recovered along with some Epipaleolithic-Upper Paleolithic (UP) artifacts^53^. AHs IIa and IIb mainly consist of Epipaleolithic (Zarzian) lithic artifacts, but a radiocarbon date and a few pottery sherds have evidenced some disturbance with the upper sediments^62^. The undisturbed Pleistocene deposits start with AH III. The early UP sequence spans from AH III to IVb, and radiocarbon and OSL dates situated this entire stratigraphic complex at the range of 42–35 kyr cal. BP^50,53,62,63^. The early UP lithic artifacts recovered at Ghar-e Boof constitute the assemblage type used for defining the Rostamian cultural group in the southern Zagros^53^. The main characteristic of the Rostamian technocomplex is the predominance of diminutive bladelets, retouched bladelet tools, and small platform cores made on radiolarian-chert cobbles^53,58,64^. Besides lithic artifacts, archaeologists also documented combustion features and personal ornaments, such as perforated shells and teeth^53,58^. Our zooarchaeological data indicate that, during the early UP, Ghar-e Boof was primarily occupied by humans and used as a campsite, while the presence of carnivores at the cave was extremely rare^65^. In addition, the faunal record shows Rostamian foragers preyed mostly on caprines for meat and marrow, but there is also evidence for the exploitation of a great variety of animal taxa, including small- to very-large-bodied ungulates (i.e., gazelles, wild pigs, red deer, equids, and wild cattle), tortoises, birds, and carnivores^65^.

AH IVd yielded an OSL date of 48–45 kyr^50^, but at present, we cannot confidently ascribe AHs IVc and IVd to either the MP or UP because of low find densities. Nonetheless, both layers lack characteristic artifacts of the UP techno-cultural repertoire in the Zagros, such as perforated shells and Arjeneh points, which were recovered in AHs IV to IVb despite having similar low find densities^50^. Consequently, AHs IVc and IVd have been tentatively assigned to either the MP^66^, or to the MP–UP transition^50^. The MP deposits have only been excavated so far in three quadrants (6/7, 6/8, and 7/7), which are located towards the central-northern part of the excavation area. Moving downwards, the OSL chronology for AHs V to Vc falls in the range of 63–46 kyr, while AH VI spans between 81 and 72 kyr (68% credible interval^50^). Although the analysis of MP lithic artifacts is still ongoing, preliminary observations indicate a technology focused on the production of flakes and diverse scrapers, in stark contrast with the UP Rostamian industries documented at Ghar-e Boof^50,59,66^. The presence of Levallois reduction techniques is currently still unclear due to the low find densities^66^. Overall, the MP record of Ghar-e Boof likely reflects short-term hominin occupations or even low populations in the Dasht-e Rostam region during the MP^58,66^. Finally, most lithic artefacts preserved sharp edges, and it seems they were recovered in their primary position.

Paleoenvironmental data inferred from the small vertebrate record of Ghar-e Boof, including small mammals, reptiles, amphibians, and fish, show that during most of the Late Pleistocene, the landscape around the site was mainly dominated by warm, arid conditions with dry, open meadows, shrublands and rocky terrain, and water sources nearby^61^. The sample size for small vertebrates is relatively small for some layers and environmental interpretations must be drawn with caution. However, the presence of Afghan pika (*Ochotona* cf. *rufescens*) in AH IVc, along with a decrease in the number of rodents between AHs IVd and IV, may suggest a short phase with slightly lower temperatures and/or drier conditions^61^. Finally, hominin remains have not been unearthed at Ghar-e Boof so far. Nonetheless, there is a general agreement among archaeologists and paleoanthropologists regarding the association of early/initial UP sites in the Zagros exclusively with Anatomically Modern Humans (AMH)s^47,50,64,66–68^, though AMH skeletal remains are very rare in the region^69–71^. As for the MP, several sites also yielded Neanderthal or Neanderthal-like remains, such as Shanidar Cave^39,72–78^, Bisotun Cave^79^, Wezmeh Cave^80^ and Bawa Yawan Rockshelter^81^. However, the complex paleoanthropological record from the MP in southwestern Asia, especially in the southern Levant, indicates early dispersals of AMHs to the region^82–84^, along with the presence of Neanderthals^85,86^. In the absence of hominin remains in MP sites, both Neanderthals and AMHs could still be considered as plausible makers of MP assemblages^50,87^.

## Data presentation and results

In this paper, we examine a sample of 941 identified specimens (Table 1; Table S2) recovered from eight layers (AHs IVc to VI) at Ghar-e Boof, ranging from ca. 81 kyr to 45 kyr^50^. Although the faunal assemblage is primarily dominated by ungulates (NISP = 710), we also present the skeletal element representation and anthropogenic modifications of carnivores (NISP = 7), tortoises (NISP = 161), and medium and large bird (NISP = 63) remains in order to assess the complete animal foraging spectrum of MP hominins at the site, beyond just ungulate/large game hunting. Our sample does not include small mammals (rodents, pikas, and insectivores), amphibians, squamate reptiles (agamid lizards and snakes) and small birds (Passeriformes), since the accumulation of these taxa at the site was most likely the result of non-hominin predator activities or natural death^61^.

**Table 1:** MP faunal assemblages from Ghar-e Boof.

AH	IVc		IVd		V		Va		Vb		Vc		Vd		VI		Total	
Taxon	NISP	%	NISP	%	NISP	%	NISP	%	NISP	%	NISP	%	NISP	%	NISP	%	NISP	%
Ungulates																		
Subtotal ungulates	56	69.1	13	76.5	71	69.6	168	69.7	98	68.1	83	71.6	28	68.3	193	97.0	710	75.5
Carnivores																		
Red fox (*Vulpes vulpes*)	0	0.0	0	0.0	0	0.0	1	0.4	0	0.0	0	0.0	0	0.0	0	0.0	1	0.1
Large carnivore	0	0.0	0	0.0	0	0.0	1	0.4	0	0.0	0	0.0	0	0.0	0	0.0	1	0.1
Leopard (*Panthera cf. pardus*)	0	0.0	1	5.9	4	3.9	0	0.0	0	0.0	0	0.0	0	0.0	0	0.0	5	0.5
Subtotal carnivores	0	0.0	1	5.9	4	3.9	2	0.8	0	0.0	0	0.0	0	0.0	0	0.0	7	0.7
Reptiles																		
Tortoise (*Testudo* sp.)	8	9.9	2	11.8	23	22.6	57	23.7	34	23.6	27	23.3	10	24.4	0	0.0	161	17.1
Birds																		
Medium birds	11	13.6	0	0.0	3	2.9	12	5.0	9	6.3	6	5.2	3	7.3	5	2.5	49	5.2
Partridge (*Alectoris* cf. *chukar*)	5	6.2	1	5.9	1	1.0	1	0.4	3	2.1	0	0.0	0	0.0	1	0.5	12	1.3
Large birds	1	1.2	0	0.0	0	0.0	1	0.4	0	0.0	0	0.0	0	0.0	0	0.0	2	0.2
Subtotal birds	17	21.0	1	5.9	4	3.9	14	5.8	12	8.3	6	5.2	3	7.3	6	3.0	63	6.7
Total	81	100.0	17	100.0	102	100.0	241	100.0	144	100.0	116	100.0	41	100.0	199	100.0	941	100.0

Number of identified specimens (NISP) and relative proportions (%) by AH for each taxon or body size group. The category “ungulates” includes all body size groups, from small to very large ungulates, which are represented by gazelle, wild sheep, wild goat, red deer, wild pig, equid, and wild cattle (for more details, see Table S2).

### Species representation

More than 75% of the MP faunal assemblage of Ghar-e Boof consists of ungulates, from small to very large taxa. The ungulate assemblage is dominated by caprines (*Ovis*/*Capra*), but mostly wild goat (*Capra aegagrus*), followed by gazelle (*Gazella* sp.). We also documented small numbers of wild pig (*Sus scrofa*), red deer (*Cervus elaphus*), equid (*Equus* sp.), and wild cattle (*Bos primigenius*). As for small game, the most common species-specific identification is tortoise (*Testudo* sp.), and species-specific designations for birds are restricted exclusively to partridge (*Alectoris* cf. *chukar*). However, the medium bird category encompasses other Galliformes and Columbiformes for which taxonomic identifications are not yet available, and large birds (small raptors) are also present. Carnivores are very rare, represented by red fox (*Vulpes vulpes*) and a large felid, probably leopard (*Panthera* cf. *pardus*).

### Skeletal element representation and bone surface modifications

Figure S1 shows the representation of each skeletal region for caprines and medium ungulates by AH (data from Table S3). Despite the sample sizes, a few patterns are evident: (1) head and limb body segments are present in all layers, and in particular, heads are the most well-represented anatomical parts; (2) we did not record any horns identified as caprine or assigned to the category “medium ungulate”; (3) neck and axial elements were not recovered from most layers, and when documented, they are visibly underrepresented; and (4) feet are also relatively rare throughout the entire MP sequence. Furthermore, correlations between skeletal elements by percentage of minimum animal units (%MAU) for all AHs combined and food utility^88^ and standard food utility^89^ indices are not statistically significant (Table S4 and S5). Instead, there are positive and statistically significant correlations between %MAU and marrow^88^ and unsaturated marrow^90^ indices respectively. Regarding the analysis of density-mediated attrition, we present ratios of ungulate tooth- to skull bone-based minimum number of elements (MNEs) by layer in Table S6. Most layers show higher cranial-based MNEs in comparison to tooth-based MNEs. There a few layers with very small sample sizes, yet they have an even ratio. Lower tooth-relative to cranial bone-based MNE values or an even ratio is the opposite of what we would expect if density-mediated attrition had influenced the faunal remains from Ghar-e Boof.

We recorded different types of bone surface modifications on the MP faunal remains recovered at Ghar-e Boof, such as sedimentological alterations, weathering and gnawing (Table S7). The most extreme damage documented in the assemblage is crushing by sediment compaction, but just less than two percent of the total remains were crushed. However, 21.2% of the total specimens were partly or completely covered by sediment concretions. Surface weathering is rare, affecting 2.5% of the bone remains. In this case, weathering damage is limited to the presence of fine linear cracks, some of them open (weathering stages 1 and 2), and none of the specimens are splintered or have surfaces with fibrous or rough textures (stages 3 to 5, after^91^). Root etching is very uncommon as well (1.1%), and none of the specimens are rounded or abraded. In contrast, chemical weathering is quite frequent, with 32.9% of the total bone specimens showing irregular etched scars and/or spots (Fig. S2). Frequencies of carnivore tooth marks and rodent gnawing are very low (1.2% and 3.0% respectively, Fig. S3; nevertheless, these frequencies would be slightly higher, 2.6% and 3.2%, if we consider specimens that were potentially gnawed, but for which the observed damage is not unequivocal).

**Figure 3:**
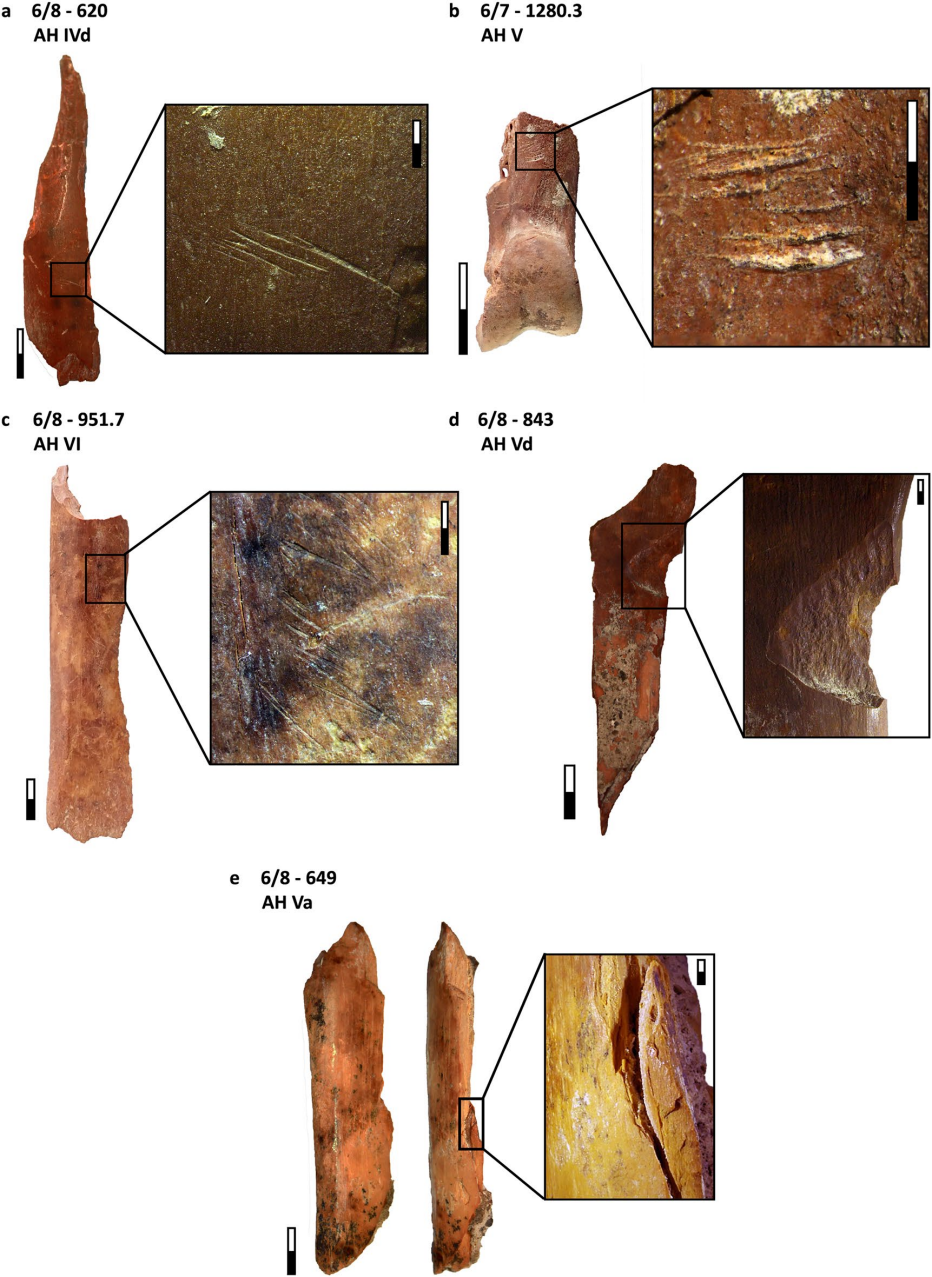
MP ungulate remains from Ghar-e Boof with anthropogenic modifications: (**a**) medium/large ungulate long bone shaft fragment with cut marks; (**b**) wild goat second phalanx with cut marks; (**c**) medium ungulate tibia heavily cut-marked; (**d**) medium ungulate tibia exhibiting an opposite cone fracture; and (**e**) medium ungulate radius with a cone fracture, in which the negative bone flake still remains attached. All these specimens also present green (split/spiral) fractures. Scale: general view = 10 mm; closer-up view = 2 mm.

Overall, burning damage occurs with fairly low intensity (Fig. S4): 6.8% of the bone assemblage is burned. Among the burned specimens, 5.8% are carbonized (stages 1 to 3) and only 1.0% are calcined (stages 4 to 6, after^92^). There is no apparent temporal change in the proportion of burned remains and burning intensity over the stratigraphic sequence. Anthropogenic modifications on bone specimens recovered at Ghar-e Boof which are associated with butchery and carcass processing activities comprise green (split/spiral and transverse) fractures, impact damage, cut marks, and bone tools (Table S8; Fig. 3). Overall, the most frequent types of damage are splits and spiral fractures, documented on more than 40.0% of the total bone assemblage. The proportion of transverse fractures, instead, is much lower (6.5% of the assemblage). Moreover, cut-marked bones are particularly abundant (20.7% of the entire assemblage), while impact damage (including cone fractures, opposite cones, and percussion impacts) is also not uncommon (8.5%). On ungulates, cut marks are more common on meat-bearing and lower limb elements (i.e., ribs, femora, humeri, radii and tibiae, Table S9). However, we also recorded cut marks on other elements, such as crania, mandibles, ulnae, metatarsals, and phalanges, and on a calcaneus, a scapula, and an astragalus. Likewise, impact damage is almost exclusively restricted to long-bone elements with high-marrow content, such as metatarsals, tibiae, femora, humeri, radii, and a mandible. One exception is an impact mark on the scapula of an aurochs, which also exhibits longitudinal scraping (Fig. S5). Finally, we identified a small number of bone retouchers (N = 6, Fig. S6), made on medium-bodied ungulate long-bone shaft fragments, along with some other potential bone tools.

**Figure 4:**
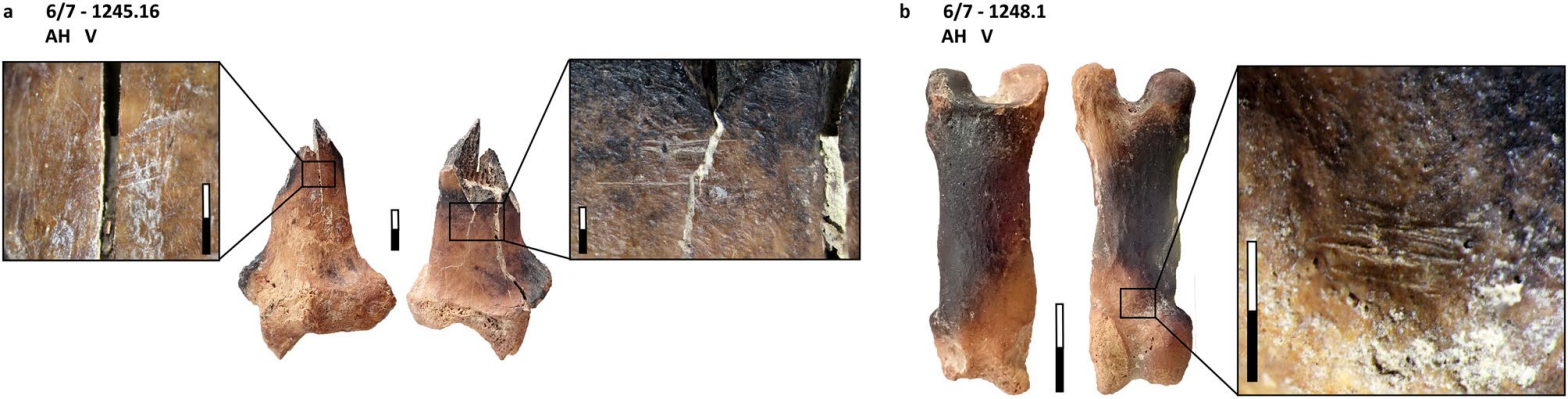
Cf. leopard remains from the MP sequence of Ghar-e Boof, which have cut marks and burning: (**a**) distal epiphysis of a radius, with cut marks located on both the anterior and posterior surfaces of the preserved shaft, and most of them are transverse, but few are also diagonal; and (**b**) first phalanx with a cluster of short and transverse cut marks in the palmar/plantar side, near the distal epiphysis.

Table 2 shows the skeletal element representation by NISP for carnivores and small game taxa. Regarding carnivores, we recorded two cranial remains: an upper molar of a red fox, and an indeterminate canine of a large carnivore (Table S10; the latter specimen was highly damaged and a more precise taxonomic identification was not possible). The rest of the carnivore bones are postcranial elements, all of them identified as cf. leopard. We documented an appendicular element (distal epiphysis of a right radius), and four complete phalanges (three first phalanges and a second phalanx). All postcranial elements are fully fused and therefore belonged to an adult individual. Despite the small number of carnivore postcranial elements in our assemblage, all of them preserve cut-marks (Table 3; Fig. 4). Moreover, a radius and two first phalanges are partially carbonized (stages 1–2), and the radius also exhibits a green fracture.

**Table 2:** MP carnivore and small game remains from Ghar-e Boof.

Taxon	Total NISP	Cranial	Axial	Appendicular	Phalanges	Shell fragments	Others
Carnivores							
Red fox (*Vulpes vulpes*)	1	1	0	0	0	NA	0
Large carnivore	1	1	0	0	0	NA	0
Leopard (*Panthera* cf. *pardus*)	5	0	0	1	4	NA	0
Reptiles							
Tortoise (*Testudo* sp.)	161	0	1	12	0	148	0
Birds							
Medium birds	49	3	9	24	12	NA	1
Partridge (*Alectoris* cf. *chukar*)	12	0	0	12	0	NA	0
Large birds	2	0	0	1	1	NA	0
Total	231	5	10	50	17	148	1

Skeletal element representation by anatomical region and NISP. All archaeological layers are combined. Data from Table S6.

*NA* not applicable.

**Table 3:** MP carnivore and small game remains from Ghar-e Boof.

Taxon	Anatomical regions	Burning	Green fractures	Percussion damage	Cut marks
Leopard	Appendicular	1 (100.0%)	1 (100.0%)	0	1 (100.0%)
	Phalanges	2 (50.0%)	0	0	4 (100.0%)
Tortoise	Axial	0	0	0	0
	Appendicular	0	0	0	2 (16.7%)
	Shell fragments	18 (12.2%)	18 (12.2%)	3 (2.0%)	2 (1.4%)
Birds	Cranial	0	0	0	0
	Axial	0	0	0	0
	Appendicular	0	6 (16.2%)	0	0
	Phalanges	0	0	0	0
	Others	0	0	0	0
Total		21 (9.1%)	25 (10.8%)	3 (1.3%)	9 (3.9%)

Anthropogenic modifications on bone specimens (by NISP and %NISP). Carnivore dental elements are not included.

**Figure 5:**
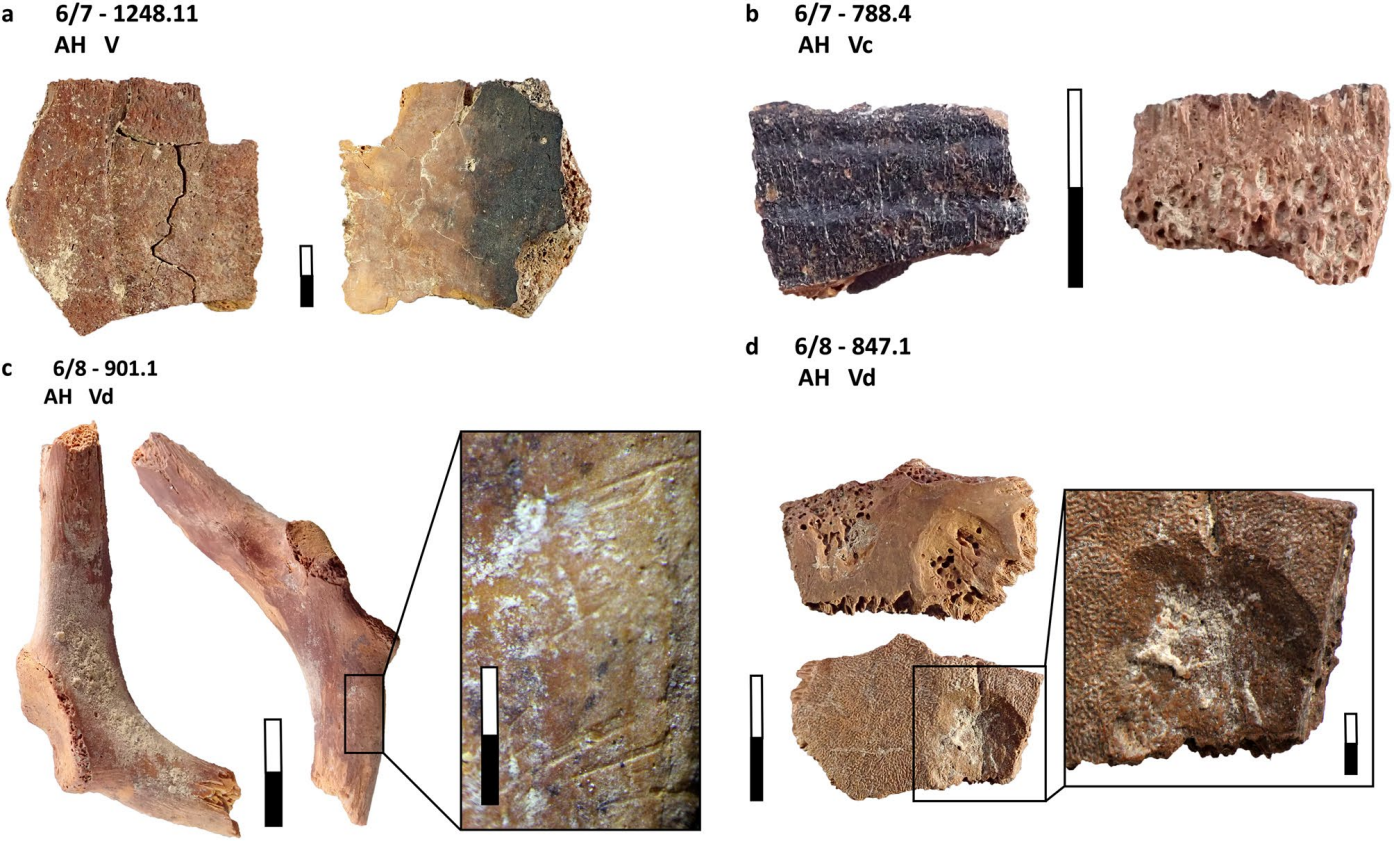
MP tortoise remains from Ghar-e Boof with anthropogenic modifications: (**a**) burnt plastron fragment; (**b**) burnt carapace shell fragment; (**c**) cut-marked scapula; (**d**) plastron fragment with a percussion impact.

The tortoise assemblage is mostly dominated by shell specimens (91.9%), comprised of both carapace and plastron fragments (Table 2; Table S10). In Fig. 5, we show some examples of tortoise specimens exhibiting burning and butchery damage. Aside from ungulates, tortoise is the taxon with the highest number of burned specimens recovered from the MP sequence of Ghar-e Boof (NISP = 18, Table 3), though burning is exclusively restricted to shell fragments. Fifteen of them were carbonized (stages 1–3), while just three were calcined (4–5). Likewise, green fractures were equally abundant and limited to carapace and plastron specimens. Other types of butchery damage were uncommon, but we documented three shell fragments with percussion impacts and another four specimens (two shell fragments and two appendicular long bones) that were cut-marked and/or scratched. Bird remains consist predominantly of long bones elements. Phalanges, cranial, and axial elements are underrepresented. None of the bird bones shows evidence of burning, and we did not find unambiguous anthropogenic modifications, such as cut marks. Green fractures are the only type of damage that might have been caused by hominins, though other agents of bone accumulation or modification (e.g., carnivores) cannot be excluded. We recorded six bird specimens (three tibiotarsi, one femur, one ulna, and one undiagnostic piece of a long bone) that exhibit either transverse or split/spiral breakages. However, the absence of carnivore damage on bird remains likewise does not allow us to rule out hominins as potential accumulators.

## Discussion and conclusions

In general, the results of our taphonomic analysis indicate that post-depositional bone surface alterations, such as weathering and root etching, are infrequent, which is expected in the protected environment of a cave^93^, and in agreement with the overall pattern documented previously in the UP sequence of Ghar-e Boof^65^. The absence of rounding or abrasion damage in our assemblage also support the hypothesis that bone specimens were found in primary position, or at least, they were not considerably displaced horizontally by water, wind or trampling. Nevertheless, a considerable proportion of the bone specimens were either partially or completely covered by sediment concretions, or affected by chemical weathering. These mineral concretions are presumably made of calcium carbonate and are most likely caused by the percolation of water through the sediments^58,65^. In our analysis, we used the term chemical weathering to describe irregular etched scars or spots on the surface of bones. The observed chemical weathering is probably the result of biological or geochemical actions^93^, for example, due to the presence of guano^60^ or the decomposition of organic materials in the burial context of the faunal remains^65^. Although both sediment concretions and chemical weathering might have obscured or even obliterated other taphonomic modifications, the relatively high proportion of green fractures, tool marks, and burning point to hominins as the main accumulating agent. Carnivore damage and rodent gnawing are rare in our assemblage, suggesting that carnivores and rodents played a minor role in the accumulation or alteration of the bones at the site. Based on the ratios of ungulate tooth- to skull bone-based MNEs, density-mediated attrition does not appear to have impacted the bone assemblage. Therefore, the faunal record of Ghar-e Boof has the potential to offer new information on hominin prey choice and subsistence strategies in the Zagros during the MP, not only including evidence for hunting and butchering ungulates, but also, at least occasionally, carnivores and small game.

During the MP at Ghar-e Boof, hominins preyed primarily on ungulates, which represent more than 75% of the identified animal specimens in the assemblage. Within the ungulate category, the most common prey was medium-bodied ungulates, including sheep/goat, but mostly wild goat. If we assume that most specimens identified as medium ungulates were most likely caprines too, then they constituted almost 60% of the animal component of the hominin diet. Hominins also hunted small ungulates such as gazelles, which are relatively abundant at Ghar-e Boof, and to a much lesser extent, we documented equid, wild pig, red deer, and wild cattle.

The analysis of skeletal element representation of caprines and medium ungulates show that our assemblage is mostly dominated by head, upper and lower limb elements, whereas horns and neck, axial and foot body segments are rare at the site. An underrepresentation of structurally weak elements, such as ribs and vertebrae, could track density-mediated attrition (e.g.,^94^). However, we feel confident that skeletal profiles mostly reflect transport practices and economic decisions for two reasons: (1) most AHs present a higher cranial-based MNEs in comparison to tooth-based MNEs; if there were density-mediated attrition, we should expect an overrepresentation of teeth, which are more resistant to attritional processes than bone due to their mineral composition^1,94^; (2) phalanges, which represent relatively dense elements^94^, are also uncommon. As a result, even if a minor degree of in-situ attrition might have occurred, we suggest that the MP occupants of Ghar-e Boof did not transport complete ungulate carcasses to the cave, abandoning in the kill sites most horns, and neck, axial and foot elements. Additionally, we have found positive and statistically significant correlations between %MAU and marrow and unsaturated marrow utility indices, which points to the preferential transportation of elements with high quantities of marrow and unsaturated fatty acids^88,90,95^ to the cave.

Overall, anthropogenic modifications on ungulate remains, such as cut marks, impact damage, and green breaks demonstrate that MP hominins exploited ungulates and butchered and processed their carcasses for meat and marrow^96–99^. Our taphonomic analysis shows that cut marks are relatively more frequent on meat-bearing and lower limb elements, including ribs, femora, humeri, radii, and tibiae. Experimental and ethnoarchaeological studies indicate that cut marks located on the above-mentioned elements are mostly associated with defleshing, filleting, and dismembering activities^96,98,99^. We also recorded cut marks on elements with relatively low utility values, which are more related to the disarticulation, skinning, and tendon removal of ungulate carcasses^96,98,99^. Moreover, most of the impact damage is located on long-bone elements with relatively high-marrow content^88,90,95^. Therefore, impact damage, along with the relatively large number of green (split/spiral and transverse) fractures, definitely suggest not only that long bones were broken when fresh through dynamic loading with hammerstones^94,97^, but also that marrow processing and the consumption of within-bone nutrients played an important economic role for MP hominins at Ghar-e Boof. Finally, we recorded six bone retouchers, which seem to have been used to retouch stone flakes.

Recently, based on the small vertebrate assemblage recovered from Ghar-e Boof, Blanco-Lapaz et al.^61^ suggested that during the Late Pleistocene the surrounding landscape of the site was mainly dominated by warm and arid conditions, open, dry meadows and shrublands, and rocky terrain, with some nearby water sources. Wild goats mostly live in piedmonts and craggy-rocky slopes, but they can also inhabit dry lowlands and steppe landscapes^100,101^. Therefore, as we suggested for the UP zooarchaeological assemblage of Ghar-e Boof^65^, the predominance of caprines and medium ungulates, along with gazelles and small-bodied ungulates, could indicate that MP hominins were likely hunting near the site, or at least locally where those environments were present. On the other hand, according to the prey choice model from optimal foraging theory, hunter-gatherers are expected to maximize their foraging efforts and energetic return rates by targeting high-ranked resources, such as ungulate prey, which provide the greatest nutritional benefits per hunting episode (i.e.^5,11,12,33^, and references therein). Optimality models predict that foragers would only turn to low-return animal resources when high-ranked prey decline or are no longer available^12,33^. If we consider caprines as high-ranked prey in the Zagros Mountains in comparison to other types of resources (e.g., small, fast-moving game), then a narrow diet, with a focus on caprines, would mean that MP hominin groups in the region were able to meet most of their daily caloric demands with high-ranked prey^5^. This narrow economic focus on medium ungulates during the MP could only have been maintained due to short-term, sporadic hominin occupations at the site, or low population densities^3,5^. In fact, our team previously suggested that Ghar-e Boof was occupied ephemerally by hominins based on the low find densities recovered from the MP deposits^58,66^, which consequently may also reflect low population densities or small population groups living in the southern Zagros region during the MP.

Regarding small game, the documentation of anthropogenic marks and burning damage on tortoise specimens demonstrate that hominins collected and processed tortoises for dietary purposes. We observed no signs of carnivore or raptor damage. Instead, green fractures and percussion impacts indicate that tortoise shells were broken and crushed with stone tools, while cut marks and scratches are associated with the defleshing and removal of meat, viscera, and ligaments^14,15^. The observed burning pattern on tortoise remains does not seem to be caused by accidental exposure to fire: (1) we only documented burning damage on shell fragments, and it appears to be restricted, with a few exceptions, to the outside surfaces of the shells, which most archaeologists have interpreted as evidence for in-shell roasting of tortoises on a fire^13,15,42,102^; (2) tortoise specimens display higher instances of burning compared to other taxa^13^; and (3) some of the shell fragments are also calcined, which is rarely accidental, since calcination of faunal remains takes place with direct exposure to live coals^92^. In addition, based on the relative abundance of tortoises throughout most of the MP sequence of Ghar-e Boof, we suggest that tortoises most likely constituted important dietary supplements. Although in general small game animals yield relatively low return rates, slow or sessile small game taxa, such as tortoises, are very easy to collect, and therefore, represent high-ranked resources because of their low capture costs^5,11,12^. The relative proportion of tortoises seems to vary little throughout time, or at least, there is no visible chronological trend. Since tortoises are susceptible to human overexploitation due to their slow maturation rates and population recovery^5,11,12^, it seems that the MP hominins that inhabited Ghar-e Boof did not exert great harvesting pressure on tortoises, which again could point to an ephemeral occupation of the site or small population groups in the region.

Carnivore remains are uncommon in the MP sequence of Ghar-e Boof. We only recorded an upper molar of a red fox, an indeterminate canine of a large carnivore, and five postcranial elements (one radius and four phalanges), which were identified as cf. leopard. Despite the small number of carnivore specimens in the assemblage, all the postcranial elements exhibit anthropogenic modifications, demonstrating the hominin exploitation of carnivores at Ghar-e Boof during the MP. These postcranial remains were recovered in a relatively small area and in close proximity to each other between the upper part of AH V, and the bottommost part of AH IVd (see Table S2 and comments). In addition, all these specimens are completely fused and have similar sizes. They probably belonged to a single adult individual, which indicates that the exploitation of carnivore resources at Ghar-e Boof represents a rare and isolated event. The observed butchery pattern on the radius and phalanges is consistent with the damage caused during disarticulation and skinning actions^103,104^, and therefore, we suggest the processing and use of carnivore pelts by MP foragers at the site. Burning damage on carnivore remains could be related to the direct exposure of bones to fire after carcass processing^104^, or to a pelt discarding event^105^, though, in this case, we cannot rule out indirect or accidental burning (e.g.,^92^). Hominins could have had access to carnivore fur through active hunting or scavenging of recently dead animals^29^. If the latter was the case, skinning for fur retrieval is a task that humans can only accomplish shortly after an animal dies (from ca. an hour to maximum of one day depending on climatic conditions), or the hair will “slip” from the hide and it will be ruined^29,106,107^. The small sample size of carnivore remains and the absence of meat-bearing elements in the assemblage potentially biases our interpretation toward skin procurement, but we do not exclude the possibility that MP foragers, on occasion, consumed carnivore meat, since it seems unlikely that they would waste fresh meat due to its nutritional value^107^. In any case, the documentation of both carnivore tooth marks on ungulate remains and anthropogenic modifications on carnivore bones recovered at Ghar-e Boof provide compelling evidence for direct and indirect carnivore-hominin interactions in the southern Zagros Mountains.

Finally, any interpretations regarding the main agent responsible for the accumulation of birds must be made with caution. In our study, we did not find unequivocal evidence for an anthropogenic origin of the bird remains recovered from the MP sequence of Ghar-e Boof, yet the most common species at the site are those that tend to be exploited by hominin foragers. Most of the bird specimens identified in the assemblage correspond to medium-sized birds, probably Columbiformes or Galliformes. However, more precise taxonomic identifications (either genus or species) were not possible for several reasons: some specimens do not present diagnostic features; there is a high diversity of birds currently distributed across modern-day Iran and southwestern Asia^108^; and we only had access to few Iranian taxa in our comparative collection. Columbiformes, such as rock doves and other pigeons, inhabit stony and rocky environments, and even breed and shelter inside caves^108^. It is possible that some bird remains come from animals that died naturally in the cave, as suggested recently for some of the small vertebrate taxa recovered at the site^61^. However, Galliformes, particularly Chukar partridges, do not live in caves^108^. A small number of medium bird or partridge specimens exhibit green fractures, indicating that the bones might have been broken by predators. On the one hand, we did not record carnivore damage on bird remains, which allows us to consider hominins as potential accumulators. On the other hand, experimental studies have demonstrated that humans can deflesh and disarticulate bird carcasses using their bare hands without the assistance of any tools^109^. Thus, the absence of cut marks on bird specimens at Ghar-e Boof does not necessarily mean that MP hominins did not exploit or consume bird resources occasionally. Following the prey choice model, small, fast-moving, or difficult-to-catch animals, such as partridges, usually provide lower caloric return rates because they have higher capture and processing costs than large game or small, slow-moving taxa^5,11,12,35^. Under the purported low population densities and hunting pressures attested in the southern Zagros during the MP, hominins were able to have a narrow diet dominated by high-ranked food resources, while low-ranked birds might have been either completely ignored or represented very sporadic contributions to the total meat intake^3,5,11,12^.

During the last decades, the reconstruction of dietary and subsistence strategies of MP hominins have benefited considerably from the improvement and standardization of modern excavation techniques (e.g., systematic water-sieving of sediments or use of ≤ 2 mm mesh, which allow archaeologists to recover even the smallest faunal remains^22,25^). In addition, there have been an increasing number of detailed taphonomic-oriented analyses that have focused on different classes of faunal remains present in archaeological assemblages (i.e.^15,19,21,22,25^). Some scholars have proposed that Neanderthals habitually exploited small, fast-moving animals, such as leporids and birds^18–20,22,24^. However, the systematic use of small, quick animals is still rare in the MP record of Eurasia^3,22^, and it might only have been a feasible foraging strategy at some localities due to their unique environmental conditions or the availability of small prey^20,22,25,30,34^. In most cases, the zooarchaeological evidence points to sporadic use of small, fast-moving game and carnivores^21,23,28^. Nevertheless, the acquisition and exploitation of diverse prey highlight the high plasticity, variability, and complex foraging skills of MP hominins throughout Eurasia.

The majority of examples of hominin utilization of diverse types of prey during the MP, especially small, fast-moving taxa, have been documented at southwestern European sites^18–26,30^. Overall, the available data are comparatively scarce in southwestern Asia, and most of the evidence comes from the northern and southern Levant. Besides a narrow focus on large game hunting, in this region, MP hominins favored the exploitation of small, slow-moving game species, such as shellfish, land tortoise, and freshwater turtle^2,3,11–14,110,111^. Our analysis of the MP faunal assemblages of Ghar-e Boof shows a similar picture. MP hominins probably met most of their meat and marrow demands with large game animals, while sessile tortoises could be considered important dietary supplements. Instead, birds and carnivores seem to have played a much more marginal role within the animal fraction of the hominin diets. If we use the observed diet breadth as an indicator of hunting pressure and demography^5,11,12^, in southwestern Asia, from the eastern rim of the Mediterranean Sea to the southern Zagros Mountains, hominin population densities were consistently low during most of the MP on local and regional scales.

In the Zagros Mountains, archaeologists have published zooarchaeological data for the following Late Pleistocene sites with MP deposits (Fig. 1): Eshkaft-e Gavi^48^, Kunji Cave^45,48^, Kaldar Cave^47^, Warwasi Rockshelter^41^, Ghar-e Khar^43^, Kobeh Cave^4,44–46,48^, and Shanidar Cave^39,40,42,49^. As we mentioned above, the only instance of purported hominin harvesting of tortoises in the region might be Shanidar Cave, on the basis of burning damage and relative species abundances^42^. Other than that, zooarchaeologists have suggested that during the MP hominins targeted ungulates across the Zagros almost exclusively, mostly caprines, along with equids and gazelles, but also red deer, roe deer, wild pig, and wild cattle^4,39,40,42–49^. Overall, the relative species abundances from these MP sites and our analysis are very similar, and a narrow focus on ungulates could reflect both environmental constraints and prey availability^48^, as well as socioeconomic decisions linked to the optimization of energetic return rates by targeting primarily high-ranked resources^5,11,12^.

Our taphonomic analysis of the MP faunal remains from Ghar-e Boof suggests that tortoises constituted important dietary supplements for hominins, and the occupants of the site only exploited carnivores and possibly birds on occasion. Nonetheless, these results still offer new insights on the diversity and flexibility of foraging behaviors of MP hominins in the Zagros Mountains. Previously, Evins^42^ suggested that the lack of tortoises at some Late Pleistocene sites might just attest to local prey availability. However, tortoises (*Testudo* spp.) are flexible in their habitat requirements, and nowadays they can be found throughout the entire range of the Zagros Mountains^112^. Chukar partridges, for example, also inhabit a great variety of habitats across the Zagros, including stony foothills, bush-covered plains, barren terrains and gullies and wadis in arid plains^108^. The ecological flexibility of these two species leads us to suggest that different classes of small game taxa must have been available in the surrounding environments of the above-mentioned sites. Likewise, the taxonomic and taphonomic analyses of the remains recovered at Bisotun Cave, Tamtama Cave, and Wezmeh Cave confirmed these sites were mainly carnivore dens^48,113^. Carnivore remains were also documented at Kobeh Cave and Eshkaft-e Gavi^48^, indicating that carnivores were either constant threats, potential resources for hominins, or both. Up to now, it seems that our understanding of hominin diets and subsistence strategies in the Zagros have been partly biased because zooarchaeologists have paid more attention to large game animals^4,43–46,48,49^. In addition, scholars have demonstrated that archaeologists who originally excavated MP sites during the 1950s or 1960s introduced certain biases because excavation standards were different compared to modern practices, they did not have access to technology such as total stations, or did not retain all the faunal remains^48,49^. Recently, new archaeological research projects have focused on re-excavating well-known sites, such as Shanidar Cave^76,77^, but also excavating relatively new sites, including Kaldar Cave^47^, Ghar-e Boof^58,59^ and Bawa Yawan Rockshelter^81^, among others, with modern excavation techniques. Further investigations are still required, but the complete recovery of even the smallest faunal remains from these excavations will almost certainly provide us with new evidence to reconstruct more exhaustively the hominin foraging spectrum, and to assess the socioeconomic importance of different types of game during the MP in the Zagros. Meanwhile, the faunal remains from the MP sequence of Ghar-e Boof represent the first evidence of hominin exploitation of small game and carnivores in the southern Zagros Mountains. Even if the use and consumption of some of these taxa were sporadic, our results demonstrate that MP hominins exploited more diverse animals than previously thought in the Zagros region, and is more consistent with what is found in other parts of Eurasia.

## Methods summary

Archaeologists excavated Ghar-e Boof by 50 cm sub-squares within each squared meter, and in two to three cm-deep spits or *abträge*, following the slope of the sediments^58^. Both lithic artifacts and organic remains were recorded in three dimensions, along with the orientation for elongated finds^58^. In order to allow the retrieval of even the smallest animal remains, excavators floated all the sediments and water-screened them using superimposed five- and two-mm mesh^58^. We identified taxonomically and anatomically the faunal remains recovered from Ghar-e Boof with the help of the zooarchaeological reference collection of the University of Tübingen. When needed, our identifications were also assisted by osteological atlases and zooarchaeological guides (i.e., for caprines^114,115^; for carnivores^116^; and for tortoises^117^; among others), in combination with other unpublished electronic manuals and images. Number of identified specimens (NISP) is the basic counting unit in this study for estimating taxonomic abundance following Grayson^118^’s and Lyman^94,119^’s definitions. Here, NISP not only includes specimens identified to the lowest possible taxonomic level, such as species, genus, or family, but also fragments with less diagnostic features that we assigned to body size groups (e.g., medium bird or small carnivore^2^). Specimens were recorded following Stiner^120^’s coding system for skeletal elements and portions of elements, with some minor modifications for Aves and Testudines. Although our zooarchaeological analysis is based exclusively on species representation and abundance comparisons by NISP counts and percentages of NISP, we estimated the minimum number of individuals (MNI) and provided them in Table S2, in order to allow comparisons with other Late Pleistocene faunal collections from the Zagros region for which MNI values are available (e.g.,^42,113^). For the quantification of MNI, we considered the most common element, as well as side and age, by taxon^94,118,119^, and for each AH.

In order to examine anatomical part representation for the main prey (caprines and medium ungulates), we grouped skeletal elements into nine body regions, which constitute logical portions in terms of butchery and transport decisions^121^. Following Binford^122^ and Stiner^121^, we calculated MAU by dividing our MNE values by the expected MNE in a complete animal skeleton for each different element and body region. We also complement our analysis of transport and butchery practices with Spearman's rank-order correlation test to examine possible relationships between %MAU (MAUs divided by the highest observed MAU value in our assemblage and then multiplied by 100^119^) and food utility^88^, standard food utility^89^, marrow^88^, and unsaturated marrow^90^ indices. Due to small sample sizes, all the caprine and medium ungulate elements recovered from MP sequence were combined. Bone density-mediated attrition was examined by contrasting ungulate tooth- and skull bone-based MNE counts^1^. Considered as a single transportable unit, the head region encompasses both bony and tooth elements that are expected to be brought together to a site, and therefore, the ratio between the most abundant tooth and bone cranial element based on MNE should be nearly 1:1^1^. Because of the differences in mineral composition and structural density, teeth better withstand attritional processes than bones^1,94^. As a result, an overrepresentation of teeth would indicate that density-mediated attrition had affected a faunal assemblage^1^. We combined all ungulate taxa together for each layer in this analysis in order to have a more robust dataset^65^.

We analyzed bone surface modifications and fractures to identify the main agent of accumulation or modification of the MP deposits of Ghar-e Boof, and to evaluate other post-depositional processes that may have affected the integrity and the interpretative protentional of the archeofaunal assemblage. All bone specimens were examined with a 10× hand lens. When the analysis of bone surface modifications required higher resolution examination and for photographing the specimens, we used an Olympus SZX7 stereo microscope with a digital camera and a Keyence VHX-500FD digital microscope, which offer magnifications from 4× to 336×, and from 5× to 200× respectively. We distinguished between physical, abiotic, and non-human biological alterations, and anthropogenic modifications. As for non-human alterations, we recorded for each specimen the presence/absence of weathering damage (e.g., cracks, flaking, and exfoliation), root etching, rounding/abrasion, chemical weathering, sedimentological alterations (sediment concretions and crushing), carnivore damage (e.g., tooth marks, crenulation, digestion, and punctures) and rodent gnawing. Our identification of such modifications follows the criteria described by^91,93,97^. Anthropogenic modifications documented for this study include burning, percussion damage, cut marks, bone tools, and green/fresh fractures (following^92–94,97^, and references therein). Among the fresh fractures, we differentiated between transverse fractures (those that occur when a bone was broken at a right angle or perpendicular to its long axis) and splits or spiral fractures (which break the bone parallel to the long axis, though spiral fractures also present a helical shape around the circumference of the bone shaft^94,123,124^. Finally, our interpretation of the different anthropogenic modifications and bone fractures (or their absence) are mainly based on ethnographic and experimental studies for ungulate^95,96,98,99^, carnivore^103,104^, and bird^109^ carcasses, and the corresponding observed butchery behaviors, such as skinning, defleshing, dismemberment, and marrow processing.

### Supplementary Information

Supplementary Information.

## Data Availability

All the data supporting the results and interpretations reported in this paper are available within the main text, figures, and tables or as Supplementary Information. For access to the zooarchaeological assemblages from Ghar-e Boof temporarily housed at the University of Tübingen, the readers may contact the co-directors of the site (N.J.C. and M.Z.).
