# Supplementary material for: Evidence of diverse animal exploitation during the Middle Paleolithic at Ghar-e Boof (southern Zagros)
Source: Sci Rep. 2023 Nov 3;13:19006. doi: 10.1038/s41598-023-45974-8 (PMC10624823; doi:10.1038/s41598-023-45974-8)
Supplement: Supplementary file 1 — Supplementary Information. [file 41598_2023_45974_MOESM1_ESM.pdf]

SUPPLEMENTARY INFORMATION

Evidence of diverse animal exploitation during the Middle Paleolithic at Ghar-e Boof (southern Zagros)

Mario Mata-González[1,2*], Britt M. Starkovich[1,3], Mohsen Zeidi[3,4], and Nicholas J. Conard[1,3,4]

[1]Institute for Archaeological Sciences, University of Tübingen, Hölderlinstr. 12, 72074 Tübingen, Germany

[2]Department of Anthropology, University of Connecticut, Unit 1176, 354 Mansfield Road, Storrs, CT, 06269, USA.

[3]Senckenberg Centre for Human Evolution and Paleoenvironment (SHEP), Hölderlinstr. 12, 72074 Tübingen, Germany

[4]Department of Early Prehistory and Quaternary Ecology, University of Tübingen, Schloss Hohentübingen, D-72070 Tübingen, Germany

*Corresponding author:

E-mail address: mario.mata-gonzalez@student.uni-tuebingen.de

Table S1. Ghar-e Boof. Summary of available stratigraphic information, including sedimentological characteristics, thickness, dating and cultural affiliations by archaeological horizon (AH). Table from Blanco-Lapaz et al.[1] and references therein.

AH	Sedimentological characteristics	Thickness	Dates (yr. BP)	Cultural affiliations
I	Mixed, gray, ashy silts of the surface and subsurface	5 to 20 cm	-	Historical periods (Late Sassanid and Early Islamic)
II	In-situ laminated ashy silt layers of black, red, orange, yellow, gray, white and various brown hues	100 cm		
IIa	Dark brown/gray ashy silts	15 cm		Mainly Epipaleolithic (Zarzian), but pottery sherds still present
IIb	Medium brown ashy silts to a light, gray/brown silts. Massive geogenic layer (IIb.1) of limestone cobles located at the bottom of this AH	10 cm	1,225-1,260 cal.	
III	Homogenous light gray to yellow-brown ashy silts, similar to loess. Ample lateral variation	10 to 70 cm	35,152 ± 368 cal.	Early UP (Rostamian)
IIIa	Medium brown ashy silts	20 cm	-	
IIIb	Light brown ashy silts with alternating gray-black, white-brown, and light brown silts	60 cm	38,994 ± 1419 cal.<br>39,949 ± 921 cal.	
IIIc	Light brown silts, starting with an irregular cemented crust	20 cm	-	
IV	Light brown silts with small angular fragments of limestone	25 cm	41,355 ± 326 cal.	
IVa	Brown silts with small angular fragments of limestone	15 cm	40 – 42 k	
IVb	Light brown silts, but less rocky than IV and IVa	15 cm	-	
IVc	Fine rocky brown silts	10 cm	-	MP – UP Transition (?)
IVd	Fine rocky light brown silts	15 cm	45 – 48 k	
V	Light brown but less rocky silts, underlaid by a thin dark brown band and then by a medium brown silty sediment	40 cm	46 – 50 k	MP
Va	Mostly light brown silty matrix with many small sharp-edged pieces of limestone, though thin bands of brown silts were observed	60 cm	51 – 55 k<br>55 – 59 k	
Vb	Still mostly rocky light brown silty matrix, along with red brown silts	25 cm	56 – 60 k	
Vc	Light brown silts	15 cm	59 – 64 k	
Vd	Light brown to yellow brown silts	25 cm	63 – 70 k	
VI	Homogeneous brown to gray-light yellow brown silts, with small limestone clasts, laying over the bedrock	80 cm	72 – 78 k<br>74 – 81 k	

Table S2. MP faunal assemblages from Ghar-e Boof. Number of identified specimens (NISP) and minimum number of individuals (MNI) by AH for each taxon or body size group.

**AH**	*IVc*		*IVd*		*V*		*Va*		*Vb*		*Vc*		*Vd*		*VI*		*Total*	
Taxon	NISP	MNI	NISP	MNI	NISP	MNI	NISP	MNI	NISP	MNI	NISP	MNI	NISP	MNI	NISP	MNI	NISP	MNI
**Ungulates**																		
Small ungulate	0	NA*	0	NA	4	NA	17	NA	11	NA	5	NA	0	NA	15	NA	52	NA
Gazelle (*Gazella* sp.)	2	1	0	0	1	1	1	1	1	1	0	0	6	1	6	1	17	6
Small/medium ungulate	2	NA	1	NA	2	NA	9	NA	11	NA	9	NA	4	NA	16	NA	54	NA
Medium ungulate	35	NA	7	NA	52	NA	111	NA	57	NA	36	NA	16	NA	112	NA	426	NA
Sheep (*Ovis* sp.)	0	0	0	0	0	0	2	1	4	1	0	0	0	0	0	0	6	2
Wild goat (*Capra aegagrus*)	10	1	0	0	4	1	2	1	1	1	7	1	0	0	9	2	33	7
Sheep/goat (*Ovis/Capra*)	2	1	3	1	4	1	11	1	9	1	23	2	1	1	26	1	79	9
Medium/large ungulate	0	NA	2	NA	0	NA	4	NA	0	NA	0	NA	1	NA	3	NA	10	NA
Large ungulate	3	NA	0	NA	3	NA	10	NA	2	NA	2	NA	0	NA	2	NA	22	NA
Red deer (*Cervus elaphus*)	1	1	0	0	0	0	0	0	0	0	0	0	0	0	0	0	1	1
Wild pig (*Sus scrofa*)	0	0	0	0	0	0	0	0	0	0	0	0	0	0	1	1	1	1
Equid (*Equus* sp.)	0	0	0	0	0	0	0	0	1	1	0	0	0	0	0	0	1	1
Large/very large ungulate	1	NA	0	NA	0	NA	0	NA	0	NA	1	NA	0	NA	1	NA	3	NA
Very large ungulate	0	NA	0	NA	1	NA	1	NA	1	NA	0	NA	0	NA	0	NA	3	NA
Wild cattle (*Bos primigenius*)	0	0	0	0	0	0	0	0	0	0	0	0	0	0	2	1	2	1
**Carnivores**																		
Red fox (*Vulpes vulpes*)	0	0	0	0	0	0	1	1	0	0	0	0	0	0	0	0	1	1
Large carnivore	0	NA	0	NA	0	NA	1	NA	0	NA	0	NA	0	NA	0	NA	1	NA
Leopard (*Panthera* cf. *pardus*)	0	0	1	1	4	1	0	0	0	0	0	0	0	0	0	0	5	2(1)**
**Reptiles**																		
Tortoise (*Testudo* sp.)	8	1	2	1	23	1	57	2	34	1	27	1	10	1	0	0	161	8
**Birds**																		
Medium birds	11	NA	0	NA	3	NA	12	NA	9	NA	6	NA	3	NA	5	NA	49	NA
Partridge (*Alectoris* cf. *chukar*)	5	2	1	1	1	1	1	1	3	1	0	0	0	0	1	1	12	7
Large birds	1	NA	0	NA	0	NA	1	NA	0	NA	0	NA	0	NA	0	NA	2	NA
**Total**	81	7	17	4	102	6	241	8	144	7	116	4	41	3	199	7	941	46(45)**

*NA (Not Applicable).

**Although we have documented Leopard's remains in AHs IVd and V, all of them were recovered relatively close to each other, just in the transition between these 2 layers, within an interval of 7 cm in depth. Therefore, they may belong to just 1 individual, instead of 2.

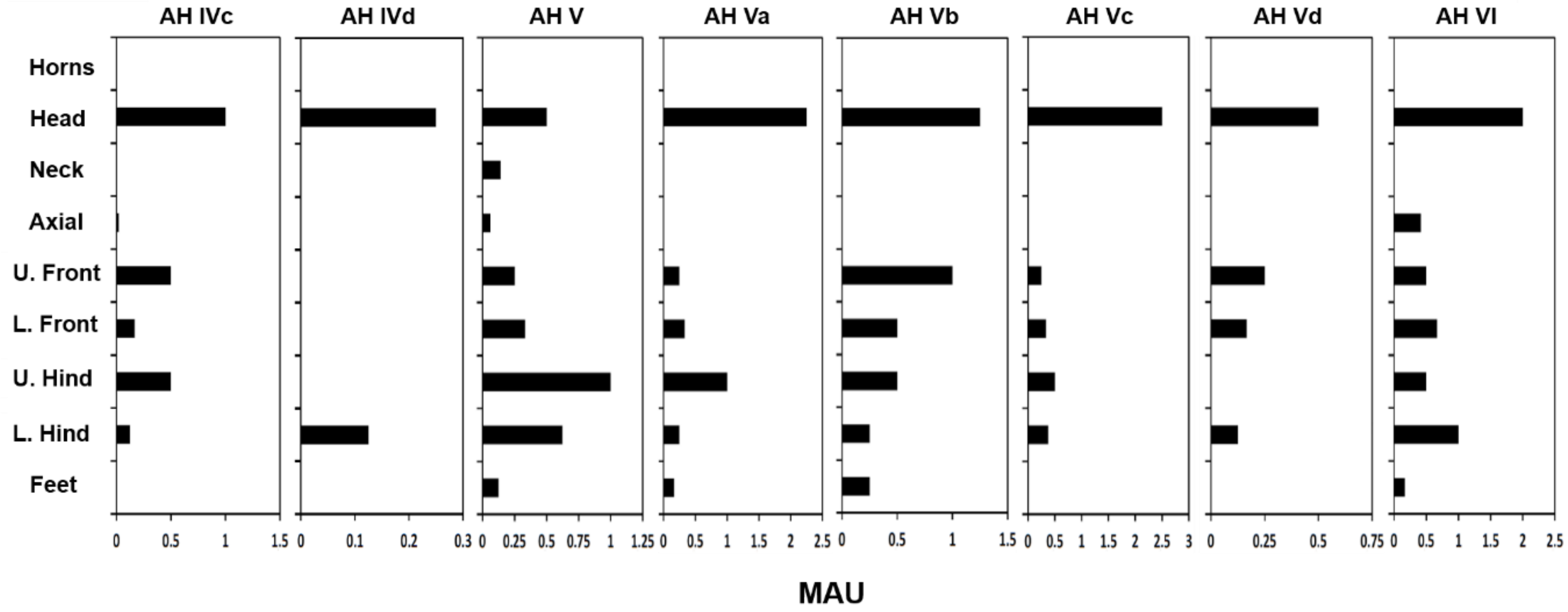

Fig. S1. MP faunal assemblages from Ghar-e Boof. MAU values for each skeletal region of caprines/medium ungulates by AH. Data from Table S4.

Table S3. MP faunal assemblages from Ghar-e Boof. NISP, MNE and MAU counts for caprine/medium ungulate skeletal elements and by AH. As a comparison, the data are presented along with a standard goat skeleton (*). Horizontal lines indicate the elements that encompasses each anatomical region.

	ST*	IVc			IVd			V			Va			Vb			Vc			Vd			VI		
Elements	MNE	NISP	MNE	MAU	NISP	MNE	MAU	NISP	MNE	MAU	NISP	MNE	MAU	NISP	MNE	MAU	NISP	MNE	MAU	NISP	MNE	MAU	NISP	MNE	MAU
Horn	2	0	0	0.0	0	0	0.0	0	0	0.0	0	0	0.0	0	0	0.0	0	0	0.0	0	0	0.0	0	0	0.0
1/2 Cranium	2	4	3	1.5	1	1	0.5	1	1	0.5	8	7	3.5	5	4	2.0	8	8	4.0	2	2	1.0	8	7	3.5
1/2 Mandible	2	1	1	0.5	0	0	0.0	2	1	0.5	2	2	1.0	2	1	0.5	4	2	1.0	0	0	0.0	5	1	0.5
Atlas	1	0	0	0.0	0	0	0.0	0	0	0.0	0	0	0.0	0	0	0.0	0	0	0.0	0	0	0.0	0	0	0.0
Axis	1	0	0	0.0	0	0	0.0	0	0	0.0	0	0	0.0	0	0	0.0	0	0	0.0	0	0	0.0	0	0	0.0
Cervical	5	0	0	0.0	0	0	0.0	1	1	0.2	0	0	0.0	0	0	0.0	0	0	0.0	0	0	0.0	0	0	0.0
Thoracic	13	0	0	0.0	0	0	0.0	0	0	0.0	0	0	0.0	0	0	0.0	0	0	0.0	0	0	0.0	0	0	0.0
Lumbar	6	0	0	0.0	0	0	0.0	1	1	0.17	0	0	0.0	0	0	0.0	0	0	0.0	0	0	0.0	1	1	0.2
Sacrum	1	0	0	0.0	0	0	0.0	0	0	0.0	0	0	0.0	0	0	0.0	0	0	0.0	0	0	0.0	0	0	0.0
Rib	26	3	1	< 0.1	0	0	0.0	1	1	< 0.1	0	0	0.0	0	0	0.0	0	0	0.0	0	0	0.0	1	1	< 0.1
Innominate	2	0	0	0.0	0	0	0.0	1	1	0.5	0	0	0.0	0	0	0.0	0	0	0.0	0	0	0.0	0	0	0.0
Scapula	2	0	0	0.0	0	0	0.0	0	0	0.0	0	0	0.0	1	1	0.5	0	0	0.0	0	0	0.0	0	0	0.0
Humerus	2	6	2	1.0	0	0	0.0	3	1	0.5	5	1	0.5	5	3	1.5	2	1	0.5	2	1	0.5	6	2	1.0
Radius	2	1	1	0.5	0	0	0.0	5	1	0.5	12	1	0.5	3	1	0.5	1	1	0.5	1	1	0.5	9	2	1.0
Ulna	2	0	0	0.0	0	0	0.0	3	1	0.5	2	1	0.5	2	1	0.5	1	1	0.5	0	0	0.0	4	1	0.5
Metacarpal	2	0	0	0.0	0	0	0.0	0	0	0.0	0	0	0.0	1	1	0.5	0	0	0.0	0	0	0.0	1	1	0.5
Femur	2	1	1	0.5	0	0	0.0	5	2	1.0	11	2	1.0	1	1	0.5	1	1	0.5	0	0	0.0	6	1	0.5
Patella	2	0	0	0.0	0	0	0.0	0	0	0.0	0	0	0.0	0	0	0.0	0	0	0.0	0	0	0.0	0	0	0.0
Tibia	2	4	1	0.5	0	0	0.0	9	3	1.5	14	1	0.5	7	1	0.5	7	2	1.0	2	1	0.5	18	5	2.5
Astragalus	2	0	0	0.0	0	0	0.0	0	0	0.0	0	0	0.0	0	0	0.0	0	0	0.0	0	0	0.0	0	0	0.0
Calcaneum	2	0	0	0.0	0	0	0.0	1	1	0.5	0	0	0.0	0	0	0.0	0	0	0.0	0	0	0.0	2	2	1.0
Metatarsal	2	0	0	0.0	1	1	0.5	1	1	0.5	3	1	0.5	1	1	0.5	1	1	0.5	0	0	0.0	3	1	0.5
Phalanx 1	8	0	0	0.0	0	0	0.0	3	2	0.3	1	1	0.1	4	3	0.4	0	0	0.0	0	0	0.0	4	2	0.3
Phalanx 2	8	0	0	0.0	0	0	0.0	2	1	0.1	4	3	0.4	2	2	0.3	0	0	0.0	0	0	0.0	2	2	0.3
Phalanx 3	8	0	0	0.0	0	0	0.0	0	0	0.0	2	2	0.3	1	1	0.1	0	0	0.0	0	0	0.0	0	0	0.0
Total	107	20	10	-	2	2	-	39	19	-	64	22	-	35	21	-	25	17	-	7	5	-	70	29	-

Table S4. MP faunal assemblages from Ghar-e Boof. NISP, MNE, MAU and %MAU for caprines/medium ungulates by element (for a more robust sample, we combined all the MP AHs), alongside MNEs for a standard goat skeleton (ST goat), Foot Utility Index (FUI; from[2]), standard Food Utility Index (sFUI; from[3]), Marrow Index (MI; from[2]), and Unsaturated Marrow Index (UMI; from[4]).

Elements	MNE ST Goat	NISP	MNE	MAU	%MAU	FUI	sFUI*	MI	UMI
Horn	2	0	0	0.0	0.0	NA**	1.0	NA	NA
Cranium***	2	37	33	16.5	100.0	25.3	9.1	NA	NA
Hemimandible	2	16	8	4.0	24.2	NA	31.1	NA	NA
Atlas	1	0	0	0.0	0.0	9.1	10.2	0.0	NA
Axis	1	0	0	0.0	0.0	9.1	10.2	0.0	NA
Cervical	5	1	1	0.2	1.2	38.6	37.1	0.0	NA
Thoracic	13	0	0	0.0	0.0	47.4	47.3	0.0	NA
Lumbar	6	2	2	0.3	1.8	45.1	33.1	0.0	NA
Sacrum	1	0	0	0.0	0.0	NA	NA	NA	NA
Rib	26	5	3	0.1	0.6	62.3	51.6	0.0	NA
Sternum	1	0	0	0.0	0.0	32.4	66.6	0.0	NA
Innominate	2	1	1	0.5	3.0	34.7	49.3	3.9	NA
Scapula	2	1	1	0.5	3.0	27.5	44.7	1.3	NA
Humerus	2	29	11	5.5	33.3	27.5	40.8	79.8	22.8
Radius	2	32	8	4.0	24.2	19.2	23.0	58.9	26.3
Ulna	2	12	5	2.5	15.2	NA	NA	NA	NA
Metacarpal	2	2	2	1.0	6.1	6.5	8.1	17.3	19.6
Carpals	12	3	3	0.3	1.8	10.7	12.7	0.0	0.9
Femur	2	25	8	4.0	24.2	100.0	100.0	87.0	34.0
Patella	2	0	0	0.0	0.0	NA	NA	NA	NA
Tibia	2	61	14	7.0	42.4	57.7	53.5	100.0	51.1
Astragalus	2	0	0	0.0	0.0	30.0	27.7	0.0	0.9
Calcaneum	2	3	3	1.5	9.1	30.0	27.7	0.0	2.6
Metatarsal	2	10	6	3.0	18.2	16.1	17.5	21.1	46.5
Tarsals	8	1	1	0.1	0.6	30.0	27.7	0.0	0.9
Phalanx 1	8	12	8	1.0	6.1	8.8	8.6	3.5	3.7
Phalanx 2	8	10	8	1.0	6.1	8.8	8.6	3.5	1.8
Phalanx 3	8	3	3	0.4	2.4	8.8	8.6	3.5	0.9

*We evaluated long bones as whole elements, so for sFUI we averaged the values provided by Metcalfe and Jones[3] for distal and medial epiphyses.

**NA (Not Applicable).

***MNE values for crania were also estimated for both left and right sides separately, and that is why the “cranium” of a standard goat skeleton has “2 MNEs”.

Table S5. Ghar-e Boof. Spearman's rank-order correlation values for the relationship %MAU and utility indices. Data from Table S4.

Utility indices	*n*	r_s	*p*
FUI	23	-0.070	0.751
sFUI	25	0.053	0.803
MI	22	0.864	< 0.001*
UMI	13	0.906	< 0.001*

*Asterisks indicate statistically significant correlations.

Table S6. Middle Paleolithic faunal assemblages of Ghar-e Boof. Ratios between ungulate tooth and bone-based MNE by AH. All ungulate taxa are included for a more robust dataset.

AH	Tooth MNE	Bone MNE	Tooth:bone MNE
IVc	2	4	0.5
IVd	1	1	1.0
V	1	1	1.0
Va	1	10	0.1
Vb	2	9	0.2
Vc	3	10	0.3
Vd	1	2	0.5
VI	3	9	0.3

Table S7. MP faunal assemblages from Ghar-e Boof. Frequencies of sedimentological alterations, weathering damage and gnawing documented on faunal specimens by AH.

**AH**	*IVc*		*IVd*		*V*		*Va*		*Vb*		*Vc*		*Vd*		*VI*		*Total*	
**Type of damage**	NISP	%	NISP	%	NISP	%	NISP	%	NISP	%	NISP	%	NISP	%	NISP	%	NISP	%
**Sedimentological alterations**																		
None	58	71.6	16	94.1	63	61.8	185	76.8	109	75.7	66	56.9	32	78.1	196	98.5	725	77.1
Sediment concretions	23	28.4	1	5.9	39	38.2	55	22.8	33	22.9	36	31.0	9	22.0	3	1.5	199	21.2
Sediment Crushing	1	1.2	0	0.0	0	0.0	1	0.4	2	1.4	14	12.1	0	0.0	0	0.0	18	1.9
Total	81	100.0	17	100.0	102	100.0	241	100.0	144	100.0	116	100.0	41	100.0	199	100.0	941	100.0
**Weathering***																		
None	32	44.4	6	40.0	62	64.6	157	69.5	87	65.4	70	77.8	30	90.9	95	56.6	539	64.7
Fine linear cracks	0	0.0	0	0.0	0	0.0	4	1.8	0	0.0	2	2.2	0	0.0	3	1.8	9	1.1
Fine cracks, some "open"	0	0.0	0	0.0	3	3.1	3	1.3	2	1.5	0	0.0	0	0.0	4	2.4	12	1.4
Chemical weathering	39	54.2	8	53.3	32	33.3	63	27.9	44	33.1	18	20.0	3	9.1	68	40.5	275	33.0
Root etching	2	2.8	2	13.3	2	2.1	0	0.0	1	0.8	0	0.0	0	0.0	2	1.2	9	1.1
Total	72	100.0	15	100.0	96	100.0	226	100.0	133	100.0	90	100.0	33	100.0	168	100.0	833	100.0
**Gnawing***																		
None	68	94.4	13	86.7	79	82.3	221	97.8	131	98.5	88	97.8	33	100.0	153	91.1	786	94.4
Carnivore bite marks	1	1.4	0	0.0	3	3.1	0	0.0	0	0.0	0	0.0	0	0.0	6	3.6	10	1.2
Potential carnivore bite marks	1	1.4	0	0.0	3	3.1	2	0.9	1	0.7	0	0.0	0	0.0	5	3.0	12	1.4
Rodent gnawing	2	2.7	2	13.3	12	112.5	3	1.3	1	0.7	1	1.1	0	0.0	4	2.4	25	3.0
Potential rodent gnawing	0	0.0	0	0.0	0	0.0	0	0.0	0	0.0	1	1.1	0	0.0	1	0.6	2	0.2
Possibly digested	0	0.0	0	0.0	0	0.0	0	0.0	1	0.7	0	0.0	0	0.0	0	0.0	1	0.1
Total	72	100.0	15	100.0	96	100.0	226	100.0	133	100.0	90	100.0	33	100.0	168	100.0	833	100.0

*Tooth elements are excluded from these analyses.

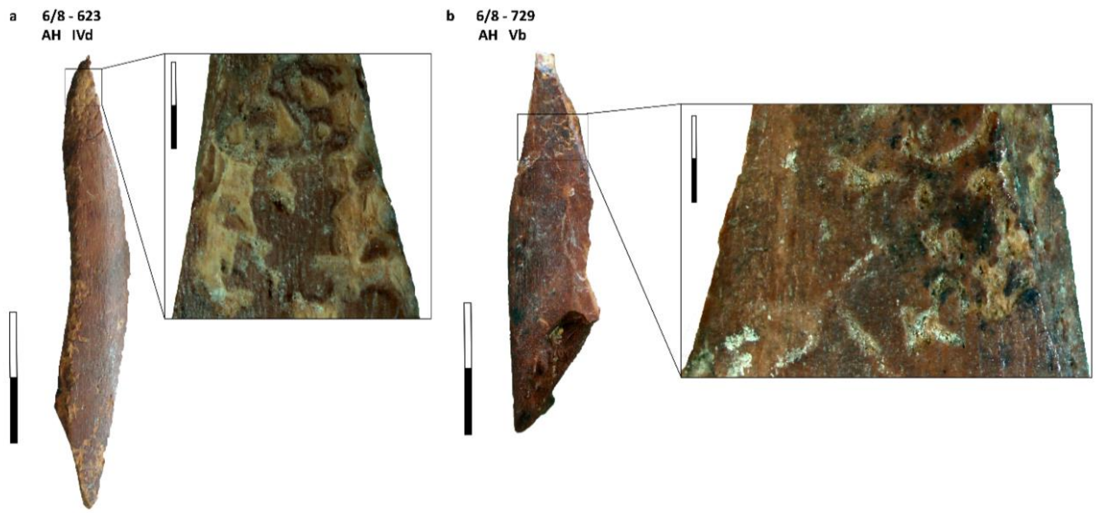

Fig. S2. MP faunal remains from Ghar-e Boof exhibiting chemical weathering: a) medium-bodied ungulate long bone shaft fragment; and b) medium ungulate tibia. Scale: general view = 10 mm; closer-up view = 2 mm.

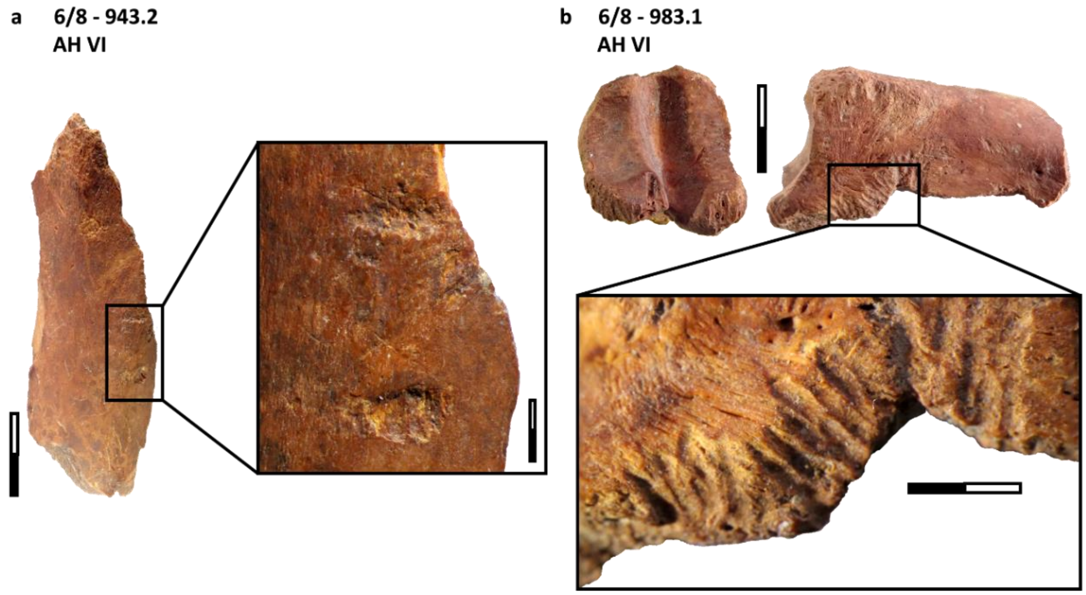

Fig. S3. MP faunal remains from Ghar-e Boof: a) medium-bodied ungulate long bone shaft fragment with carnivore bite marks; b) wild goat first phalanx with rodent gnawing. Scale: general view = 10 mm; closer-up view = 2 mm.

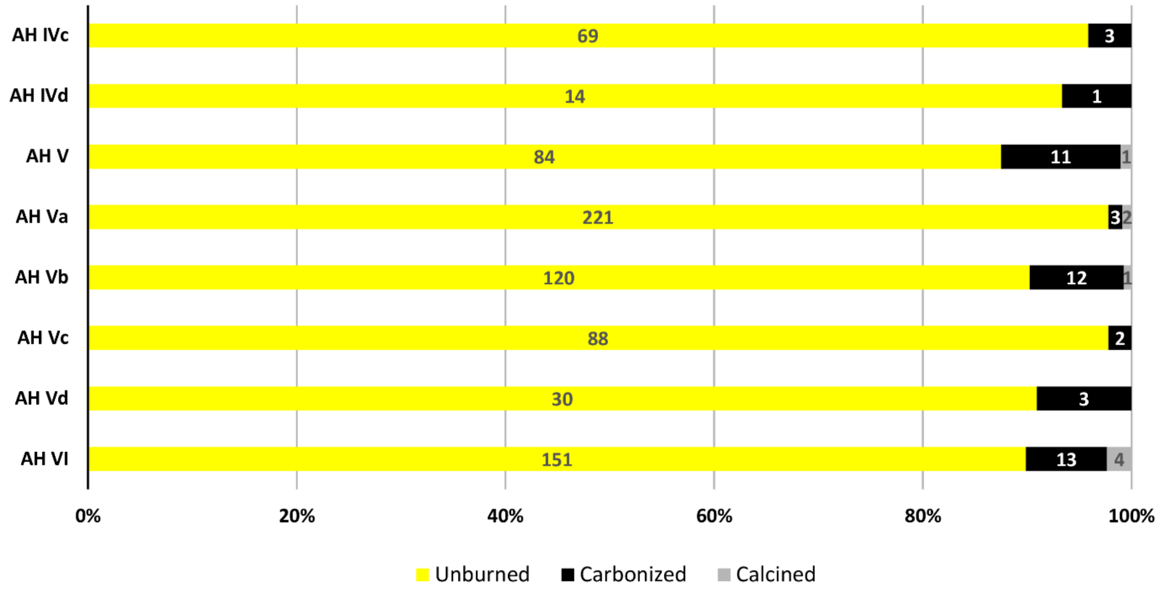

Fig. S4. MP faunal assemblages from Ghar-e Boof. NISP count and %NISP of unburned and burned (carbonized/calcined) bones by AH.

Table S8. MP faunal assemblages from Ghar-e Boof. Butchery damage on faunal specimens by AH. Tooth remains are excluded.

**Butchery damage**	*IVc*		*IVd*		*V*		*Va*		*Vb*		*Vc*		*Vd*		*VI*		*Total*	
	NISP	%	NISP	%	NISP	%	NISP	%	NISP	%	NISP	%	NISP	%	NISP	%	NISP	%
Impact damage	8	11.1	1	6.7	3	3.1	16	7.1	8	6.0	9	10.0	6	18.2	20	11.9	71	8.5
Potential impact damage	0	0.0	0	0.0	0	0.0	0	0.0	0	0.0	0	0.0	2	6.1	1	0.6	3	0.4
Transverse fracture	6	8.3	0	0.0	8	8.3	10	4.4	14	10.5	5	5.6	0	0.0	11	6.6	54	6.5
Split/spiral fracture	26	36.1	8	53.3	33	34.4	98	43.4	47	35.3	35	38.9	10	30.3	89	53.0	346	41.5
Cut marks	6	8.3	4	26.7	19	19.8	48	21.2	21	15.8	11	12.2	4	12.1	57	33.9	170	20.7
Potential cut marks	0	0.0	0	0.0	0	0.0	0	0.0	1	0.8	1	1.1	1	3.0	3	1.8	6	0.7
Bone tools	1	1.4	0	0.0	0	0.0	2	0.9	2	1.5	0	0.0	0	0.0	1	0.6	6	0.7
Potential bone tools	0	0.0	0	0.0	1	1.0	3	1.3	0	0.0	0	0.0	0	0.0	2	1.2	6	0.7
**Total**	72	100.0	15	100.0	96	100.0	226	100.0	133	100.0	90	100.0	33	100.0	168	100.0	833	100.0

Table S9. MP ungulate remains (NISP) from Ghar-e Boof. NISP values, and specimens with cut marks (CMs) and percussion (P) damage (N) by element and body size groups (all MP layers combined). In order to provide a more robust sample, the relative proportions (%) of anthropogenic modifications by element are shown only for all the ungulate remains combined, including specimens assigned to intermediate categories (e.g., small/medium ungulate). Tooth specimens are excluded.

	**Small ungulate**			**Medium ungulate**			**Large ungulate**			**Very-large ungulates**			**Total**				
**Elements**	NISP	CMs	P	NISP	CMs	P	NISP	CMs	P	NISP	CMs	P	NISP	CMs	%	P	%
**Cranial**																	
Horn	1	0	0	0	0	0	0	0	0	0	0	0	4	0	0.0	0	0.0
Cranium	4	0	0	37	1	0	0	0	0	0	0	0	52	3	5.8	0	0.0
Mandible	2	1	1	20	3	0	0	0	0	1	0	0	27	5	18.5	1	3.7
Hyoid	0	0	0	1	0	0	1	0	0	0	0	0	2	0	0.0	0	0.0
Subtotal	7	1	1	58	4	0	1	0	0	1	0	0	85	8	9.4	1	1.2
**Axial**																	
Atlas	0	0	0	0	0	0	0	0	0	0	0	0	0	0	0.0	0	0.0
Axis	0	0	0	0	0	0	0	0	0	0	0	0	0	0	0.0	0	0.0
Cervical vertebra	0	0	0	1	0	0	0	0	0	0	0	0	1	0	0.0	0	0.0
Thoracic vertebra	0	0	0	0	0	0	0	0	0	0	0	0	3	0	0.0	0	0.0
Lumbar vertebra	0	0	0	2	0	0	0	0	0	0	0	0	3	0	0.0	0	0.0
Rib	2	1	0	4	0	0	1	0	0	0	0	0	11	3	27.3	0	0.0
Innominate	0	0	0	1	0	0	0	0	0	0	0	0	1	0	0.0	0	0.0
Subtotal	2	1	0	8	0	0	1	0	0	0	0	0	19	3	15.8	0	0.0
**Appendicular**																	
Scapula	0	0	0	1	1	0	0	0	0	1	0	1	2	1	50.0	1	50.0
Humerus	3	1	1	29	6	3	0	0	0	0	0	0	33	8	24.2	5	15.6
Radius	4	2	0	32	9	4	1	1	0	0	0	0	38	12	31.6	4	10.5
Ulna	0	0	0	12	1	0	0	0	0	0	0	0	12	1	8.3	0	0.0
Carpals	0	0	0	3	0	0	0	0	0	0	0	0	4	0	0.0	0	0.0
Metacarpal	3	0	0	2	1	0	0	0	0	0	0	0	5	1	20.0	0	0.0
Femur	4	2	1	24	9	3	0	0	0	1	1	1	29	12	41.4	5	17.9
Patella	0	0	0	0	0	0	0	0	0	0	0	0	0	0	0.0	0	0.0
Tibia	2	1	0	61	16	10	3	1	1	1	0	1	69	18	26.1	13	18.8
Tarsals	0	0	0	1	0	0	0	0	0	0	0	0	1	0	0.0	0	0.0
Astragalus	0	0	0	2	1	0	0	0	0	0	0	0	2	1	50.0	0	0.0
Calcaneus	0	0	0	1	1	0	0	0	0	0	0	0	1	1	100.0	0	0.0
Metatarsal	2	0	1	10	2	3	0	0	0	0	0	0	13	2	15.4	4	30.8
Subtotal	18	6	3	178	47	23	4	2	1	3	1	3	209	57	27.3	32	15.3

Table S9. Continued.

	Small ungulate			Medium ungulate			Large ungulate			Very-large ungulates			Total				
**Elements**	NISP	CMs	P	NISP	CMs	P	NISP	CMs	P	NISP	CMs	P	NISP	CMs	%	P	%
**Feet**																	
Sesamoid	2	0	0	25	0	0	0	0	0	0	0	0	28	0	0.0	0	0.0
First phalanx	4	0	0	12	1	0	1	0	0	0	0	0	17	1	5.9	0	0.0
Second phalanx	3	1	0	10	4	0	0	0	0	0	0	0	14	5	35.7	0	0.0
Third phalanx	1	0	0	3	0	0	0	0	0	0	0	0	4	0	0.0	0	0.0
Subtotal	10	1	0	50	5	0	1	0	0	0	0	0	63	6	9.5	0	0.0
**Others**																	
Unknown long bone	17	12	1	139	60	27	15	4	3	1	0	0	185	80	43.2	33	17.8
Flat bone	0	0	0	2	0	0	1	0	0	0	0	0	6	1	16.7	0	0.0
Unknown vertebra	0	0	0	3	0	0	0	0	0	0	0	0	4	0	0.0	0	0.0
Unknown carpal/ tarsal	0	0	0	1	1	0	0	0	0	0	0	0	1	1	100.0	0	0.0
Unknown metapodial	6	2	0	19	4	1	0	0	0	0	0	0	30	7	23.3	1	3.3
Unknown phalanx	0	0	0	1	0	0	0	0	0	0	0	0	2	0	0.0	0	0.0
Subtotal	23	14	1	165	65	28	16	4	3	1	0	0	228	89	39.0	34	14.9
**Total**	60	23	5	459	121	51	23	6	4	5	1	3	604	163	27.0	67	11.1

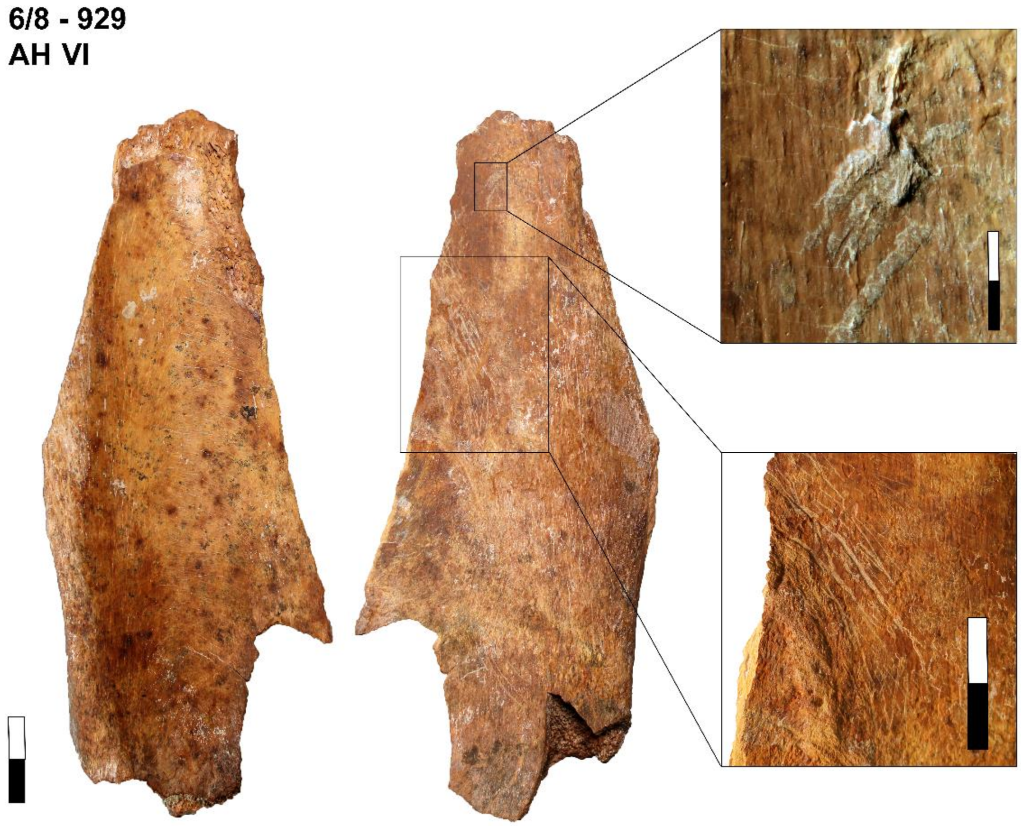

Fig. S5. Scapula of an aurochs recovered from the MP sequence of Ghar-e Boof, which has a percussion impact and longitudinal scraping. Scale: general view = 20 mm; closer-up view = 2 mm.

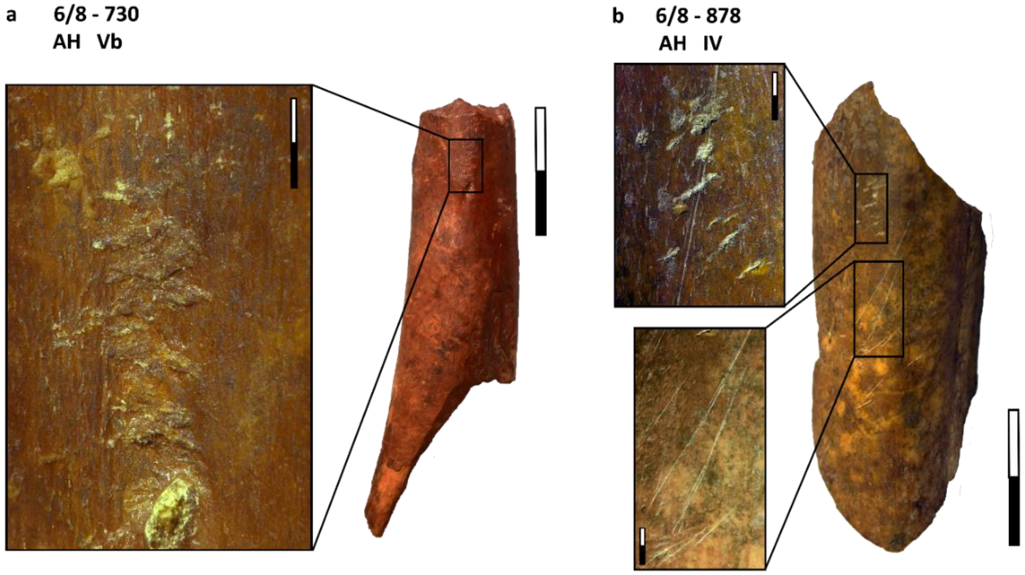

Fig. S6. Bone retouchers from the MP sequence of Ghar-e Boof: a) medium ungulate humerus with retouching marks; and B) medium ungulate femur with cut marks/scraping and retouching marks. Scale: general view = 10 mm; closer-up view = 2 mm.

Table S10. MP carnivore and small game remains from Ghar-e Boof. Skeletal elements (data in NISP) by taxon or body size group (all layers combined)

**Elements**	**Carnivores**			**Tortoise**	**Birds**			**Total**
	Red fox (Vulpes vulpes)	Large carnivore	Leopard (*Panthera* cf. *pardus*)	Tortoise (*Testudo* sp.)	Medium birds	Partridge (*Alectoris* cf. *chukar*)	Large birds	Total
**Cranial**								
Cranium	0	0	0	0	3	0	0	3
Indet. canine	0	1	0	NA*	NA*	NA*	NA*	1
Upper molar	1	0	0	NA*	NA*	NA*	NA*	1
Subtotal	1	1	0	0	3	0	0	5
**Axial**								
Axis	0	0	0	0	1	0	0	1
Cervical vertebra	0	0	0	0	3	0	0	3
Thoracic vertebra	0	0	0	0	1	0	0	1
Pelvic girdle	0	0	0	1	2	0	0	3
Furculum	NA*	NA*	NA*	NA*	2	0	0	2
Subtotal	0	0	0	1	9	0	0	10
**Appendicular**								
Coracoid	NA*	NA*	NA*	1	1	1	0	3
Scapula	0	0	0	1	1	0	0	2
Humerus	0	0	0	5	1	2	0	8
Radius	0	0	1	0	1	1	0	3
Ulna	0	0	0	0	2	1	0	3
Carpometacarpus	NA*	NA*	NA*	NA*	3	1	0	4
Femur	0	0	0	2	3	1	0	6
Tibia	0	0	0	2	NA*	NA*	NA*	2
Tibiotarsus	NA*	NA*	NA*	NA*	5	4	1	10
Fibula	0	0	0	0	1	0	0	1
Tarsometatarsus	NA*	NA*	NA*	NA*	4	1	0	5
Indet. long bone	0	0	0	1	2	0	0	3
Subtotal	0	0	1	12	433	12	1	50
**Phalanges**								
1st phalanx	0	0	3	0	11	0	0	14
2nd phalanx	0	0	1	0	0	0	1	2
3rd phalanx	0	0	0	0	1	0	0	1
Subtotal	0	0	4	0	12	0	1	17
**Shell fragments**								
Carapace & Plastron	NA*	NA*	NA*	148	NA*	NA*	NA*	148
**Others**								
Unknown	0	0	0	0	1	0	0	1
**Total**	1	1	5	161	49	12	2	231

NA* (Not Applicable).

References:

1. Blanco-Lapaz, A., Mata-González, M., Starkovich, B. M., Zeidi, M. & Conard, N. J. Late Pleistocene environments in the southern Zagros of Iran and their implications for human evolution. *Archaeol. Anthropol. Sci.* **14,** 161; https://doi.org/10.1007/s12520-022-01615-1 (2022).

2. Emerson, A. M. The role of body part utility in small-scale hunting under two strategies of carcass recovery in *From Bones to Behavior. Ethnoarchaeological and Experimental Contributions to the Interpretation of Faunal Remains* (ed. Hudson, J.) 138–155 (Center for Archaeological Investigation. Southern Illinois University, 1993).

3. Metcalfe, D. & Jones, K. T. A reconsideration of animal body-part utility indices. *Am. Antiq.* **53,** 486–504; https://doi.org/10.2307/281213 (1988).

4. Morin, E. Fat composition and Nunamiut decision-making: a new look at the marrow and bone grease indices. *J. Archaeol. Sci.* **34,** 69–82; https://doi.org/10.1016/j.jas.2006.03.015 (2007).
